# NPF2 is involved in intracellular pH regulation and ion balance in the diatom *Phaeodactylum tricornutum*

**DOI:** 10.1111/nph.71169

**Published:** 2026-04-21

**Authors:** Anna Santin, Monia Teresa Russo, Dany Croteau, Antonella Ruggiero, Sara Russo Spena, Laura Morales de los Rios, Claire Corratge‐Faillie, Seleem Brignone, Benoit Lacombe, Remo Sanges, Maurizio Chiurazzi, Maurizio Ribera d'Alcalà, Benjamin Bailleul, Angela Falciatore, Maria Immacolata Ferrante, Alessandra Rogato

**Affiliations:** ^1^ Stazione Zoologica Anton Dohrn Villa Comunale Naples 80121 Italy; ^2^ Institut de Biologie Physico‐Chimique UMR7141, CNRS‐Sorbonne Université rue Pierre et Marie Curie 13 Paris 75005 France; ^3^ European Molecular Biology Laboratory Meyerhofstraße 1 Heidelberg 69117 Germany; ^4^ Institute of Biosciences and BioResources, CNR Via P. Castellino 111 Naples 80131 Italy; ^5^ IPSiM Institut des Sciences des Plantes de Montpellier UMR Université de Montpellier/CNRS/INRAE/Institut Agro Montpellier, Place Viala Montpellier Cedex 34060 France; ^6^ Scuola Internazionale Superiore di Studi Avanzati (SISSA) Via Bonomea 265 Trieste 34136 Italy; ^7^ National Institute of Oceanography and Applied Geophysics Borgo Grotta Gigante Sgonico (Trieste) 34010 Italy

**Keywords:** genome editing, ion balance, low‐affinity nitrate transporters, NPFs, periplastidial compartment PPC, pH, *Phaeodactylum tricornutum*

## Abstract

- Cellular ion and pH homeostasis are crucial factors affecting and regulating metabolic reactions, protein stability, signalling and transport mechanisms. To balance intracellular ion concentrations, organisms need to constantly sense and respond to both the extracellular environment and cell compartments' requests.
- This fine‐tuned system has been deeply studied in organisms, such as land plants and animals, but for diatoms, living in a highly variable environment, the key regulators of intracellular homeostasis have not yet been characterized. Previous results showed a pH response in the expression of the *Phaeodactylum tricornutum* NRT1/PTR Family transporter 2 (*NPF2*) gene, homologous to bacterial Proton‐coupled Oligopeptide Transporters (POTs) involved in proton translocation across membranes.
- Here, the *P. tricornutum* NPF2 protein was shown to localize in the periplastidial compartment, between two of the four membranes of diatom chloroplasts, and to play a role in maintaining intracellular pH (pH_i_) homeostasis as well as in controlling photosynthetic and photo‐acclimation processes. Moreover, transcriptomic analyses comparing the expression profiles of wild‐type and a NPF2 knockout mutant strain highlighted an overall regulation of stress inducible genes, critical in maintaining redox homeostasis.
- These results allow us to propose PtNPF2 as a key player in maintaining pH_i_ homeostasis, with important implications for cellular processes that are sensitive to pH changes.

## Introduction

Maintaining ion and pH homeostasis is essential for the viability of all eukaryotic cells, as metabolism, protein stability, ion channel activity, compartments integrity, signalling and membrane trafficking events depend on tightly regulated ionic and pH conditions (Shen *et al*., 2013). Because environmental conditions often fluctuate, sustaining pH homeostasis under changing environmental conditions is thus paramount to preserve healthy metabolisms. To achieve this, intracellular pH (pH_i_) needs to be coordinated not only with the external environment but also with the internal cell physiological demands. pH_i_ indeed varies within different intracellular compartments and generates proton gradients which act as the fuel necessary to regulate specific processes and the general metabolism of the cell, securing its viability (Shen *et al*., 2013; Feng *et al*., 2020).

pH_i_ regulation involves not only proton concentration but also several ions with a buffering capacity, and proteins playing an important role in stabilizing the pH of different compartments. Moreover, pH_i_ control depends on both metabolic ions consumption and production, as well as transmembrane ion transport and compartmentalization (Taylor *et al*., 2012). In marine phytoplankton in particular, the uptake of nutrients, including inorganic forms of nitrogen (nitrate, NO_3_^−^, or ammonium, NH_4_^+^) and carbon (such as bicarbonate, HCO_3_^−^), is often coupled with the transport of ions, such as Na^+^, H^+^ or Cl^−^, which directly alters ionic and pH balance in cells (Taylor *et al*., 2012). Furthermore, metabolic processes resulting in the generation and consumption of H^+^ in marine phytoplankton include the production of large quantities of dimethylsulphonioproprionate (DMSP) from sulphate, the uptake and use of HCO_3_^−^ in the operation of carbon‐concentrating mechanisms (CCMs), and the generation of intracellular acidic compartments for silicification in diatoms, all of which shape the balance of H^+^ fluxes between cellular compartments (Taylor *et al*., 2012).

An organelle for which pH contrasts between compartments play a fundamental role is the chloroplast. The difference in pH between the plastid stroma and the thylakoid lumen creates a transmembrane pH gradient, which forms the osmotic component of the proton motive force and participates in its second component, the electric gradient. While the proton motive force drives chloroplast ATP synthesis, the pH gradient plays additional regulatory roles and is often considered as a ‘master regulator’ of photosynthesis (Lepetit *et al*., 2022), tuning electron transport rate (Laisk *et al*., 2005), photoprotection (Trinh & Masuda, 2022) and CCMs (Burlacot & Peltier, 2023).

Less explored is, however, the pH gradient across envelope membranes, caused by the difference between the alkaline chloroplast stroma (*c*. pH 8) and the nearly neutral cytosol (*c.* pH 7.1–7.5), which may affect the transport of ions and metabolites across the chloroplast membrabes through proton‐based exchangers, antiporters and symporters (Trinh & Masuda, 2022). Whereas pH‐dependent proton exchange across thylakoid membranes has been clearly linked to photosynthetic regulation (Seydoux *et al*., 2022), exchanges between chloroplast and cytosol are less studied, even if these could have strong implications for the movement of ions between compartments and for the tuning of the photosynthetic machinery in the face of fluctuating conditions. In particular, ions like H^+^ and HCO_3_^−^ need to pass through membranes to maintain delicate intracellular equilibrium. This trafficking is likely influenced by additional protein‐containing compartments and, in diatoms, the secondary endosymbiosis‐derived plastid bounded by four membranes appears to be directly involved (Maier *et al*., 2015; Flori *et al*., 2016).

Within this framework, maintaining organism‐level pH balance requires multiple actors, compensatory mechanisms and signalling pathways embedded in a complex regulatory network. Thus, how is the pH homeostasis of a diatom cell maintained and what are the molecular actors involved in this regulatory network?

A possible candidate belongs to the family of putative Low‐Affinity Nitrate Transporters, called NRT1/PTR Family transporters (NPFs) (Léran *et al*., 2014), well characterized in many organisms, such as land plants and bacteria. In these organisms, they have been shown to transport a broad range of different substrates, beside NO_3_^−^, together with H^+^ or other ions, controlling membranes' proton gradient (Fan *et al*., 2017). Diatom NPFs (diNPFs) diverge into two Clades: one closer to bacteria and the other closer to higher plants (Santin *et al*., 2021). However, diNPFs are still poorly characterized at the functional level (Santin *et al*., 2024), and considering the relatively low NO_3_^−^ concentrations in marine environments, their role as low‐affinity transporters seems unlikely, opening the possibility for alternative functions.

*Phaeodactylum tricornutum*, widely used as a model organism for genomic and genetic tools available, owns two NPFs: each belonging to one of the two clades (Santin *et al*., 2021). In particular, PtNPF2 belongs to bacteria‐like Clade I and it is phylogenetically and structurally closer to bacteria POTs, known to be H^+^‐coupled oligopeptide transporters. Also, PtNPF2 showed differential gene expression in different pH conditions (Santin *et al*., 2021). Here, by molecular and functional genetic analyses, new insight on PtNPF2 as an actor participating in the regulation of intracellular homeostasis upon sudden pH changes is provided.

## Materials and Methods

### *Phaeodactylum tricornutum* culture conditions

Axenic culture of *Phaeodactylum tricornutum* Bohlin, CCMP 632, was obtained from the Provasoli‐Guillard National Centre for Culture of Marine Phytoplankton. The culture was maintained in buffered F/2 medium without silica (Guillard, 1975), at 18°C and white light of 90 μmol photons m^−2^ s^−1^, with a 12 h : 12 h, light : dark cycle.

### Generation of *PtNPF2* overexpressing lines

The full‐length *PtNPF2* gene (Phatr3_J47218) coding sequence was fused to the *yellow fluorescence protein (YFP)* gene at its 3′ end or with *green fluorescence protein (GFP)* gene at its 5′ end. The two constructs were generated using the Gibson Assembly method (Gibson *et al*., 2009, 2010) following the manufacturer's instructions. For *PtNPF2‐YFP*, the backbone was provided by the PtLhcf2pPtovoAyfp plasmid (Russo *et al*., 2023), while the PmH4pH4N‐GFP plasmid (Sabatino *et al*., 2015) was used for *GFP‐PtNPF2*. Details of oligonucleotides are listed in Supporting Information Table S1. For periplastidial compartment (PPC) co‐localization, the pPha‐NRsHsp70_BTS_mRuby3‐NAT plasmid was kindly provided by Dr Meier (Marter *et al*., 2020). *P. tricornutum* cells were transformed as reported in Falciatore *et al*. (1999) and grown in F/2 medium supplemented with antibiotics: 50 μg ml^−1^ of phleomycin to select *PtNFP2‐YFP* and *GFP‐PtNPF2* mutants, where the target plasmid was co‐transformed with the *Sh‐Ble* resistance cassette (Falciatore *et al*., 1999), and 300 μg ml^−1^ nourseothricin to select transformants containing pPha‐NRsHsp70_BTS_mRuby3‐NAT plasmid, already containing NAT resistance. Transformed cells were tested for the presence of the transgene by colony polymerase chain reaction (PCR; see Table S1 for primer sequences).

### Confocal microscopy

Subcellular localization analysis was performed using a Leica SP8 X Confocal Laser‐Scanning Microscope, using the HC PL APO CS2 63X/1.20 water objective. Chl *a* autofluorescence was excited at 554 nm and detected at 680–740 nm. YFP/GFP fluorescence was excited at 488 nm and detected at 500–562 nm, while mRuby3 fluorescence was excited at 488 nm and detected between 570 and 610 nm, in a sequential mode to minimize the risk of the presence of residual YFP/GFP signal in the mRuby3 detection channel (Marter *et al*., 2020). Hoechst 33342 (Life Technologies) was used at a final concentration of 5 μg ml^−1^ to stain nuclear DNA; stained cells were visualized by excitation at 405 nm and detection at 424–462 nm.

### Generation of *PtNPF2* knockout mutants

**Fig. 1:**
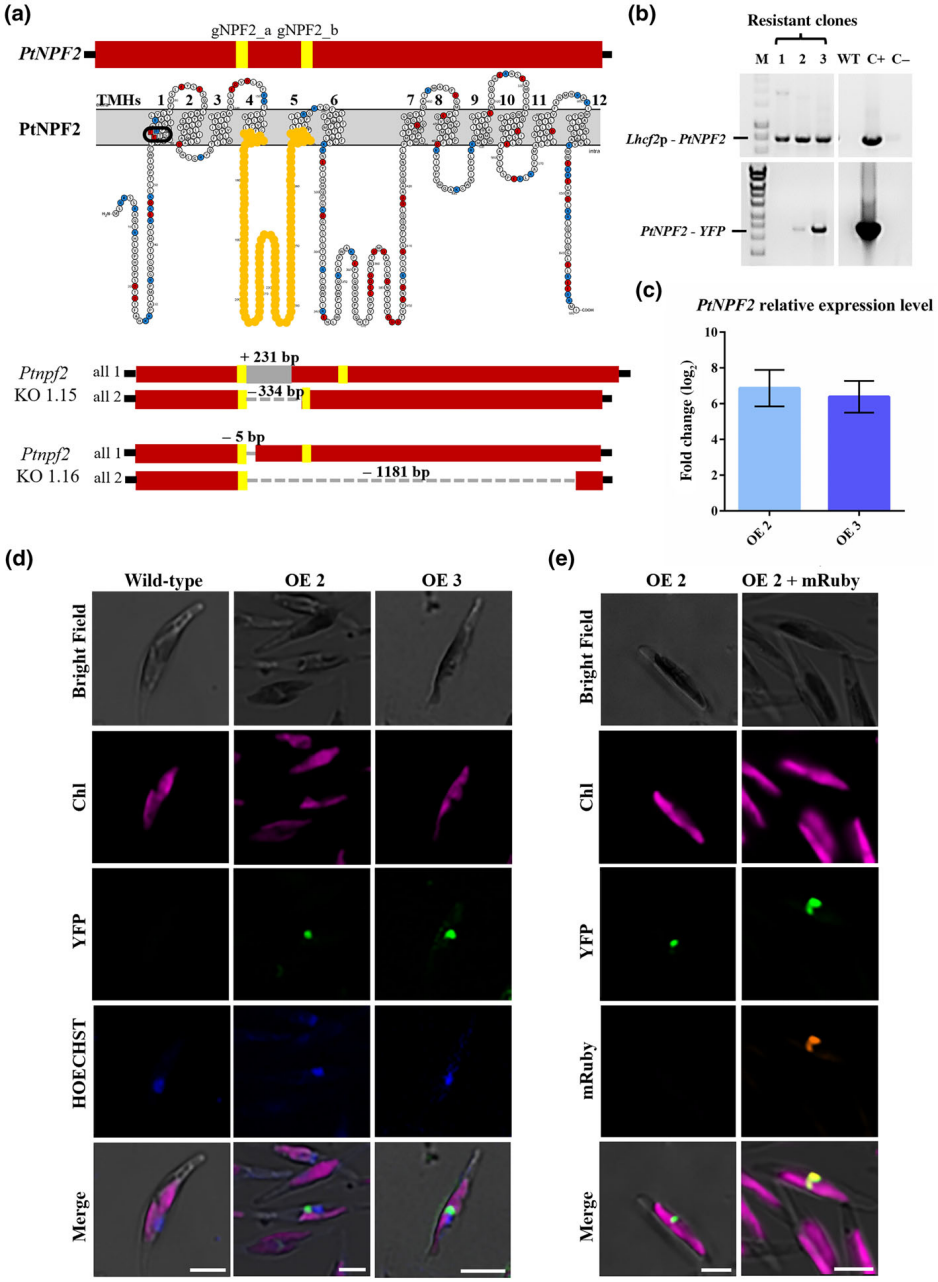
Generation of *Phaeodactylum tricornutum Ptnpf2* knockout (KO) and *PtNPF2‐YFP* overexpressing (OE) strains. (a) Guide RNA (gRNAs) designed and correspondence to transmembrane helix (TMH) 4 and TMH5 in the folded protein: yellow bars in the red *PtNPF2* gene bar indicate the gRNAs; the orange residues on the predicted PtNPF2 protein structure indicate amino acids included between the two designed gRNAs; dark cycle represents the ExxER motif (Santin *et al*., 2021). (b) Polymerisation chain reaction (PCR) showing amplification of a fragment from promoter *Lhcf2p* to the gene of interest, *PtNPF2*, and a fragment from *PtNPF2* to *yellow fluorescence protein (YFP)*, in order to detect the whole cassette of interest inserted. Primers used are listed in Supporting Information Table S1. M represents the 1‐Kb marker, C+ the plasmid and C‐ the blank. (c) *PtNPF2* relative gene expression levels of *P. tricornutum PtNPF2‐YFP* OE strains 2 and 3, normalized on the internal control *RPS* gene and on the wild‐type, set as zero. Error bars represent the SD of three technical replicates of two biological replicates (*n* = 6). (d) Subcellular localization of PtNPF2‐YFP in *P. tricornutum*. First row: bright‐field (BF); second row: Chla autofluorescence in magenta; third row: YFP fluorescence in green; fourth row: HOECHST staining for nucleus visualization in blue; fifth row: merged channels. Bars, 5 μm. (e) Subcellular co‐localization of PtNPF2‐YFP and BTS‐Hsp70‐mRuby fusion protein in *P. tricornutum*. First row: bright‐field; second row: Chla autofluorescence in magenta; third row: YFP fluorescence in green; fourth row: mRuby localized into the periplastidial compartment (PPC) in orange; fifth row: merged channels. Bars, 5 μm.

Guide RNAs were obtained using the CRISPOR tool (http://crispor.tefor.net/) (Haeussler *et al*., 2016). The two crRNA sequences designed on *PtNPF2* (gNPF2_a and gNPF2_b) cover the 500–829‐bp region of the *PtNPF2* gene sequence, which corresponds to the 166–276‐aa region of the PtNPF2 protein sequence (Fig. 1a). This region includes two transmembrane helices (TMH4 and TMH5; Fig. 1a), in which there is one of the key residues K169, involved in the salt bridge, which stabilizes outward open conformation of the PtNPF2 protein (Santin *et al*., 2021). The crRNAs on the selective gene *PtAPT* were chosen from Serif *et al*. (2018; Table S1). *In vitro* Clustered regularly interspaced palindromic repeats (CRISPR)/Cas9 ribonucleoprotein (RNP) cleavage assay was performed as described in Russo *et al*. (2022). RNP complexes were assembled following Serif *et al*. (2018) and delivered to wild‐type (WT) cells by particle bombardment (Falciatore *et al*., 1999). Transformed colonies were lysed following Daboussi *et al*. (2014) and genotyped using Sanger sequencing (see Table S1 for primer sequences).

### pH shift experiment

For the normal to low pH shift experiment, *P. tricornutum* WT, *PtNPF2‐YFP* overexpressing (OE) strains 2 and 3, and *Ptnpf2* knockout (KO) lines 1.15 and 1.16 were grown axenically for 4 d in F/2 medium without silica, buffered at pH 8 using hydrochloric acid (HCl) (Guillard, 1975) to mid‐exponential phase (1.5–2 × 10^6^ cells ml^−1^). Then, the same inoculum was diluted 1 : 10 and split in a fresh HCl‐buffered F/2 medium at pH 8 (normal pH, as control condition) or pH 7 (low pH). The experiment was performed in triplicate. Cells were counted through a Z2 Coulter Particle Count and Size Analyser (Beckman Coulter), and photosynthetic parameters and relative pH_i_ were measured just before the shift, and 3, 24 and 48 h after the shift.

### Photosystem II parameters measurements

To measure Photosystem II (PSII) parameters, 10^6^ total cells were concentrated 10 times by centrifugation and let to recover gently agitated under low light for 20 min. Fluorescence analysis was conducted using a fluorescence charge‐coupled device (CCD) camera recorder (SpeedZen, JBeamBio, France). This fluorometer is equipped with a light‐emitting diode (LED) blue measuring light and a LED green actinic light that also provides the saturating pulse (250 ms, 532 nm and 5000 μmol photons m^−2^ s^−1^). Maximum quantum yield of PSII in the dark was calculated as *F*_v_/*F*_m_ = (*F*_m_−*F*_0_)/*F*_m_, where *F*_0_ is the minimum and *F*_m_ the maximum fluorescence detected immediately after a saturating light pulse (Genty *et al*., 1989). Samples were then exposed to increasing intensities of actinic light to construct the steady‐state light curves, each illumination step lasting 3 min or 5 min for the two first lower intensities (to ensure full activation of photosynthesis). Minimal and maximal fluorescence were measured at the end of each light step, termed *F* and $F_{m}^{\prime }$, respectively. PSII quantum yield (Φ_PSII_) for a given light intensity (*E*) was calculated as ($F_{m}^{\prime }$−F)/$F_{m}^{\prime }$ and relative electron transport rate (rETR) as Φ_PSII_ × *E*. We fitted rETR as a function light intensity as rETR = rETR_max_ × (1 − e^(−αE/rETRmax)^), where rETR_max_ is the asymptotic, maximal, value of rETR and α is the initial slope of the function. Also, it was confirmed that there were no variations in the functional antenna of PSII among strains with a miniFIRe (Gorbunov & Falkowski, 2021). Nonphotochemical quenching (NPQ) was then calculated at the end of each last step as NPQ = *F*_m_/$F_{m}^{\prime }$ − 1 and the asymptomatic, maximal, values of NPQ (NPQ_max_) as a function of *E* was derived as in (Serôdio & Lavaud, 2011).

### Intracellular pH measurements

The relative intracellular cytosolic pH value was measured mainly following Dixon *et al*. (1989) and Shi *et al*. (2019). The 4 × 10^6^ total cells of *P. tricornutum* were concentrated into 400 μl PBS 1×. A 5 μM final concentration of BCECF/AM (Molecular Probe Life Technologies, Carlsbad, CA, USA) was used. After 30 min of incubation at 37°C, fluorescence emission at the wavelength of 530 nm was measured at the excitation wavelengths of 420 and 470 nm, through using the Infinite® M1000Pro Plate Reader (Tecan). The ratio of the emission wavelengths with excitation at 470 nm to that at 420 nm was calculated and was proportional to the pH_i_ (Shi *et al*., 2019).

### Pigment analysis

Cells were concentrated 10 times by centrifugation and then let for 30 min under very low light (*c*. 1 μmol photons m^−2^ s^−1^) in a 15‐ml Falcon tube under gentle agitation. Fifty microlitres was sampled and immediately dropped in 950 μl of pure methanol, quickly vortexed and flash‐frozen in liquid nitrogen before storage at −80°C. Pigment analysis was done following (Berne *et al*., 2018) protocol, with a Shimadzu Prominence‐I LC‐2030C 3D HPLC (Shimadzu Corp., Kyoto, Japan) equipped with a Waters Nova‐Pak C18 4 μm 3.9 × 150 mm column (Waters Corp., Milford, CT, USA).

### Statistical analyses

To determine whether there were significant differences in growth, photosynthetic parameters and pH_i_ between the different strains and over time, a two‐way ANOVA with Tukey's multiple comparison test was performed, and statistics for time, strain and interaction were recorded. Pairwise *post hoc* Tukey tests were applied for all multiple comparison procedures when differences were found significant (*P* < 0.05). All statistical analyses were performed using GraphPad Prism v.10.2.1.

### RNA extraction and sequencing

WT and *Ptnpf2* KO 1.15 cells were collected 24 h after the shift to normal or low pH. Three biological replicates for each sample were collected and analysed. Around 10^8^ cells were harvested and extracted with TRIzol™ (Invitrogen), as described in Santin *et al*. (2021). RNA samples were then treated with DNase I (Qiagen) to remove the gDNA contamination, and further purified using Direct‐zol™ RNA Microprep (ZYMO Research, Irvine, CA, USA).

RNA samples were quantified and quality tested by Agilent 2100 Bioanalyzer RNA assay (Agilent technologies, Santa Clara, CA, USA); then, the TruSeq Stranded mRNA Library Kit (Illumina, San Diego, CA, USA) has been used for library preparation according to the manufacturer's instructions. Final libraries were checked with both Qubit 2.0 Fluorometer (Invitrogen) and Agilent Bioanalyzer DNA assay; then, they were sequenced in paired‐end 150‐bp mode on NovaSeq 6000 (Illumina).

Read quality was inspected using FASTQC, and no critical issues were observed (Andrews, 2010). Raw reads were mapped to the reference genome using STAR with default parameters (Dobin *et al*., 2013). Read counts were assigned to each annotated gene using HTSeq‐count with default parameters. The read counts were then loaded into the R environment, and gene expression analysis was performed using the DESeq2 package with the DESeq function with default parameters (Love *et al*., 2014). Significantly differentially expressed genes (DEGs) were selected for each comparison based on an false discovery rate‐adjusted *P*‐value < 0.05 and a log_2_ fold change > ±1. The reference genome (Rastogi *et al*., 2018) and transcriptome were downloaded from Ensembl Genomes Protists release 57 (Harrison *et al*., 2024). Genes were annotated with their InterPro annotations using BioMart from the Ensembl Genome Protist website (Kinsella *et al*., 2011). Enrichment analysis was performed by using iDEP (Ge *et al*., 2018).

Additional gene expression analyses were performed through qPCR, as described in Santin *et al*. (2021). Fold changes were obtained with the Relative Expression Software Tool‐Multiple Condition Solver (REST‐MCS; Pfaffl *et al*., 2002), *PtRPS* (Phatr3_J10847) was used as a housekeeping gene, and log_2_ fold changes above ±2 were considered significant (see Table S1 for primer sequences).

## Results

### Mutants generation and subcellular localization

To characterize the function of PtNPF2 and to determine its subcellular localization, KO mutants and OE strains of *P. tricornutum* were generated. The formers were obtained through the CRISPR‐Cas9 proteolistic method, resulting in *Ptnpf2* KO strains 1.15 and 1.16, which hold insertions and deletions on both alleles that cause frameshifts and codon‐stop insertions (Figs 1a, S1). As no signal peptide was predicted for PtNPF2, the OE strains were generated through the stable insertion in the *P. tricornutum* genome of cassettes containing the *Lhcf*2 strong promoter (Russo *et al*., 2023) and the *PtNPF2* gene in frame with a 3′‐*YFP* (or a 5′‐*GFP* as a different tag, under the control of the constitutive *PmH4* promoter; Sabatino *et al*., 2015). *PtNPF2‐YFP* OE 2 and OE 3 showed a *PtNPF2* overexpression of 6.87 ± 0.42 and 6.39 ± 0.36 log_2_ fold change, respectively (Fig. 1b,c). When *PtNPF2‐YFP* OE strains were observed through confocal microscopy, the fusion protein fluorescence appeared to be localized in a small round structure close to the nucleus (Fig. 1d). This structure was generally a single spot, but sometimes it had a bilobate shape (Fig. S2). Its position is between the nucleus, with which it does not co‐localize, and the chloroplast, generally in the central part of the cell. In particular, the one or two dot‐like fluorescence structures are near the middle of the plastid, where the lobes of the autofluorescence are constricted (Fig. 1d). This localization pattern was confirmed in different *GFP‐PtNPF2* OE strains (Fig. S2).

This localization was hypothesized to correspond to the PPC, a compartment located between the four membranes of the diatom chloroplast, which is proposed to have key roles in regulating the fluxes of molecules between cytosol and chloroplast (Flori *et al*., 2016; Maier *et al*., 2022). To confirm this hypothesis, the *PtNPF2‐YFP* OE 2 strain was transformed with a plasmid expressing the fluorescent mRuby tag downstream the signal peptide of the gene encoding a chaperone (Hsp70) experimentally localized to the PPC (Marter *et al*., 2020). This strategy enabled the use of mRuby as a reliable PPC marker. As a result, the PtNPF2‐YFP fusion protein was indeed observed to co‐localize with mRuby, thereby providing strong evidence that PtNPF2 is indeed targeted to the PPC (Figs 1d, S3).

### Impact of PtNPF2 absence on growth and photosynthesis

**Fig. 2:**
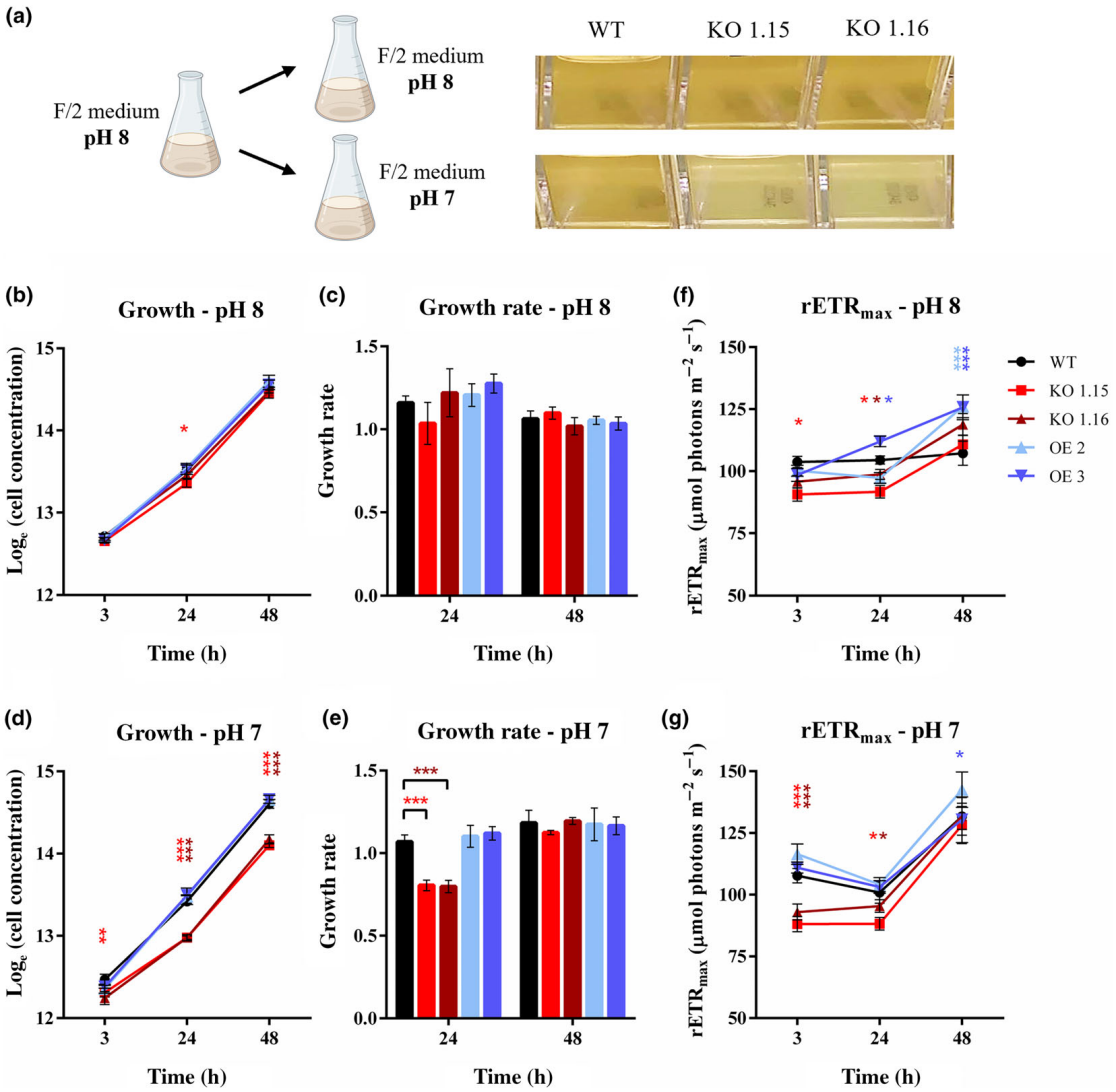
Growth curves of *Phaeodactylum tricornutum* strains after the shift from normal to low pH. (a) Schematic representation of the pH shifts experimental plan, performed in triplicate, with photographs of the liquid cultures exposed to the two different pH conditions. *P. tricornutum* wild‐type (WT), two *PtNPF2‐YFP* overexpressing (OE) strains and two *Ptnpf2* knockout (KO) mutants after the shift from pH 8 to the same pH condition (b, c, f) and to pH 7 (d, e, g), both performed in 882 μM NaNO_3_ as nitrogen (N) source. Graphs represent the natural logarithm of growth curves measured in cell concentrations of cultures grown at pH 8 (b) and pH 7 (d), growth rates of cultures grown at pH 8 (c) and pH 7 (e) and maximum relative electron transport rate, rETR_max_ of cultures grown at pH 8 (f) and pH 7 (g) as a function of time; calculated after 0, 3, 24 and 48 h from the shift. Error bars represent SD of three biological replicates. Asterisks of different colours represent significant difference (two‐way ANOVA, *n* = 3 biological replicates) between the strain of those colours and the WT. Statistical significance: *, *P* < 0.05; **, *P* < 0.01; ***, *P* < 0.001.

The phenotype of *PtNPF2‐YFP* OE and *Ptnpf2* KO strains was then studied to infer the PtNPF2 role in *P. tricornutum* cell metabolism and physiology (Fig. 2a). As *PtNPF2* showed different expression at different medium pH conditions (Santin *et al*., 2021), the effects of a low pH shift on *P. tricornutum* strains were investigated. No growth difference was observed between different strains when cells were diluted in normal pH (pH 8; Fig. 2b,c), while a reduction in *Ptnpf2* KO mutants' cell concentration was observed at low pH (pH 7; Figs 2d,e, S4; Methods S1 and), compared with WT and *PtNPF2‐YFP* OE strains. The same low pH phenotype was observed in KO strains exposed to different N sources (Fig. S4), suggesting a pH‐specific phenotype, not depending on the medium N source.

In particular, a 25% reduction in growth rate in *Ptnpf2* KO mutants was observed during the first 24 h, followed by a return to the WT growth rate after 48 h (Fig. 2e), suggesting that the phenotype is a lag in growth, rather than a stable decrease. Such transient growth phenotype was investigated in terms of photosynthetic performances, and in particular of electron transport rate. A lower maximum relative ETR (rETR_max_) was observed in *Ptnpf2* KO mutants, compared with WT, after 3 and 24 h from the shift to pH 7, which was not seen at pH 8 (Fig. 2f,g): these differences were *c*. 13–18%, in *Ptnpf2* KO strains after 3 h from the shift to pH 7, and of 5–12% after 24 h, compared with WT (Fig. 2g). Both mutants displayed a consistent phenotype, exhibiting similar and specific patterns of change (Fig. S4). OE lines, on the contrary, did not show significant differences compared with WT at both pH, except for an increase in rETR_max_ after 48 h in normal pH (Fig. 2f).

Growth curves performed at different NO_3_^−^ concentrations, using NH_4_^+^ and urea as alternative N sources, and KNO_3_ as alternative salt to NaNO_3_, showed no growth differences between *Ptnpf2* KO mutant compared with WT and *PtNPF2‐YFP* OE strains, which do not display any altered phenotype (Fig. S4), in accordance with gene expression studies not showing *PtNPF2* gene regulation in such conditions (Santin *et al*., 2024). In these experiments, which were monitored for up to 10 d, the only difference observed was a transient reduction in the growth of the *Ptnpf2* KO strains at pH 7, even when different N sources were used (Fig. S4f,h,i).

**Fig. 3:**
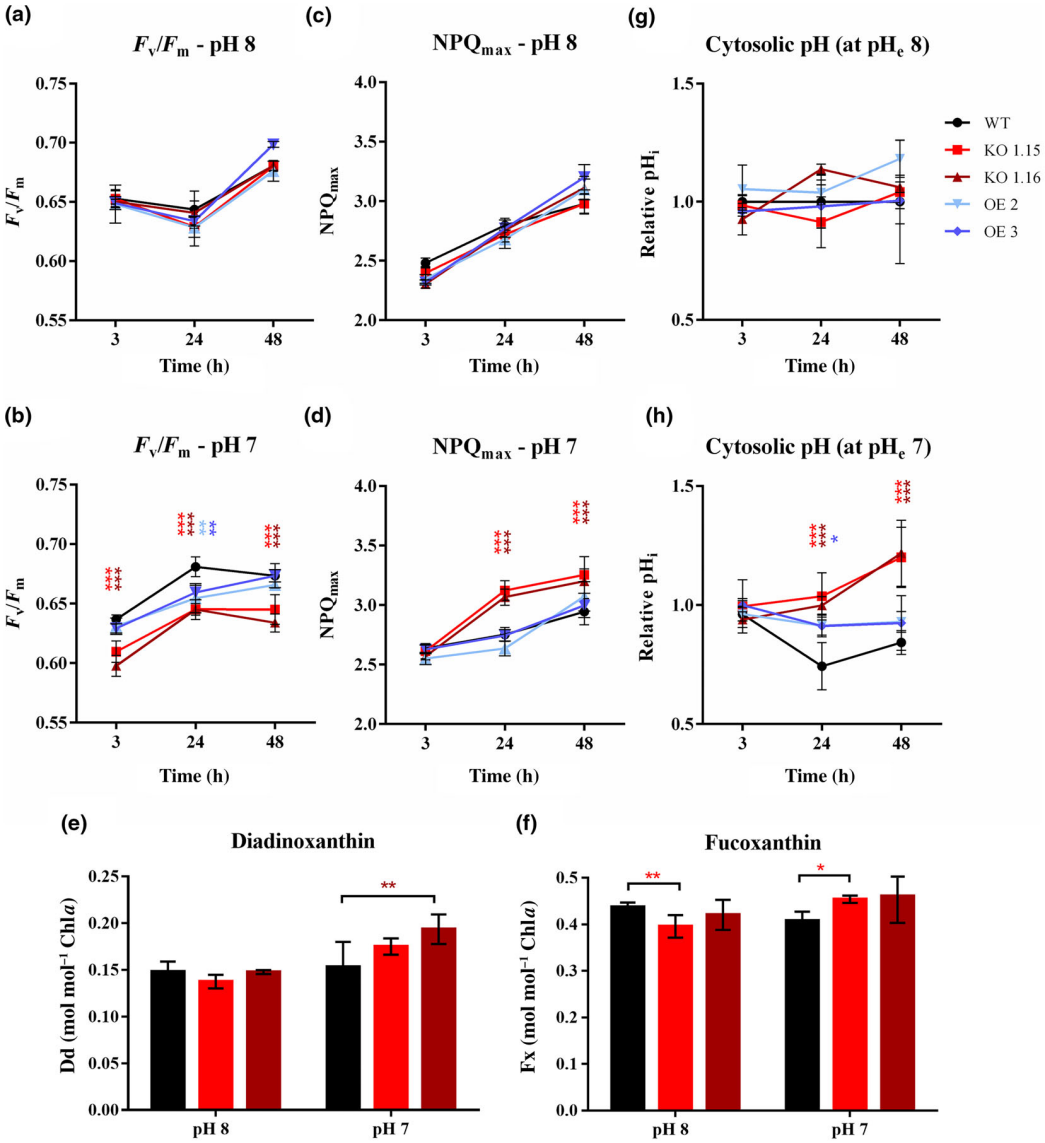
Photosynthetic and pH‐related parameters for *Phaeodactylum tricornutum* strains after the shift from normal to low pH. *P. tricornutum* wild‐type (WT), two *PtNPF2‐YFP* overexpressing (OE) strains and two *Ptnpf2* knockout (KO) mutants after the shift from pH 8 to the same pH condition (a, c, g) and to pH 7 (b, d, h), performed in 882 μM NaNO_3_. Photosynthetic measurements include yield of Photosystem II (PSII), *F*_v_/*F*_m_ (a, b) and the maximum nonphotochemical quenching, NPQ_max_ (c, d) as a function of time. Pigment analysis performed on *P. tricornutum* strains, WT and *Ptnpf2* KO mutants after 24 h from pH shift: (e) diadinoxanthin (Dd) and (f) fucoxantin (Fx) molar content, normalized on Chla molar content. pH‐related measurements are represented by the relative cytosolic intracellular pH (pH_i_), expressed as fluorescence ratio on the control condition at pH 8 and time zero (g, h). pH_e_ represents the external pH present in the medium. All these measurements, except pigment analyses, were carried out after 3, 24 and 48 h from the pH shift. Error bars represent SD (±SD) of three biological replicates. Asterisks of different colours represent significant difference (two‐way ANOVA, *n* = 3 biological replicates) between the strain of those colours and the WT. Statistical significance: *, *P* < 0.05; **, *P* < 0.01; ***, *P* < 0.001.

This transient phenotype, mainly observed in the first hours of the experiment (Fig. 2), was then further investigated in terms of photosynthetic efficiency, energy dissipation and pH_i_. The maximum dark‐adapted yield of PSII (*F*_v_/*F*_m_) was lower in *Ptnpf2* KO mutants compared with WT at all the time points after the shift to low pH but not in the normal one (Fig. 3a,b). To note that after 24 h, both *PtNPF2‐YFP* OE and *Ptnpf2* KO strains exposed to normal pH showed a decrease in *F*_v_/*F*_m_, thus suggesting a light stress induced by the dilution and followed by an acclimation recovery (Fig. 3a,b).

Then NPQ, strictly connected with the acidification of the lumenal pH, was analysed. While after the shift to normal pH, no differences have been observed between strains (Fig. 3c), after 24 and 48 h from the shift to low pH, *Ptnpf2* KO strains showed a significant increase in NPQ_max_ compared with other strains (Fig. 3d). In particular, increases of 11–13% after 24 h and of 8–10% after 48 h from the shift to low pH have been observed in *Ptnpf2* KO mutants compared with WT at the same time points and in the same pH condition (Fig. 3d). In general, NPQ response has been observed later in time (at 24 h) compared with *F*_v_/*F*_m_ and ETR responses, visible already 3 h after the pH shift. This temporal offset suggests that the *Ptnpf2* KO strains undergo distinct acclimatory phases, in which early photo‐physiological adjustments are followed by slower regulatory processes aimed at limiting light‐induced stress. These later adjustments likely involve the modulation of molecular components underpinning NPQ, including xanthophyll‐cycle pigments and their associated enzymes (Blommaert *et al*., 2021), as well as light‐harvesting complexes (LHCX) proteins (Buck *et al*., 2019).

Consistent with this, pigment analyses performed on dark‐adapted *Ptnpf2* KO mutants grown at pH 7 revealed an increase in diadinoxanthin (Dd) content, which is the substrate of the Violaxanthin de‐epoxidase (VDE) enzyme involved in NPQ activation (Figs 3e, S5). Similarly, fucoxanthin (Fx) levels were higher in *Ptnpf2* KO mutants than in WT (Figs 3f, S5), while diatoxanthin levels were below the detection threshold and β‐carotene showed no significant difference. Together, these pigment changes indicate an overall expansion of the light‐harvesting pigment and photoprotective capacity, pointing to a preacclimated or stress‐primed state in the mutants that affects pigment metabolism even under dark conditions.

Also, the relative pH_i_, indicating specifically the cytosolic one, was measured. Recent studies showed that *P. tricornutum* pH_i_ decreases as the external pH decreases, as an acclimation strategy to external pH changes (Shi *et al*., 2019). This is consistent with the relative pH_i_ trend in WT and *PtNPF2‐YFP* OE strains after the shift to low pH (Fig. 3g,h), while *Ptnpf2* KO mutants showed relative pH_i_ higher than WT after 24 and 48 h, with values more similar to those measured at normal external pH (Fig. 3g). This difference suggests an impeded capability to balance the internal pH homeostasis of *Ptnpf2* KO mutants compared with the other strains. Interestingly, no significant phenotype was identified in *PtNPF2‐YFP* OE strains (Figs 2, 3, S4), which did not differ from WT growth and photosynthetic parameters.

### Transcriptional effects of PtNPF2 mutation in cells exposed to low pH

The reduction in growth rate and impaired photosynthesis upon shift to low pH was further investigated at the molecular level. One *Ptnpf2* KO mutant (1.15) was randomly selected, together with the WT, to perform a transcriptomic analysis on the shift experiment from normal to low pH after 24 h, in three biological replicates. The principal component analysis showed that samples of both WT and *Ptnpf2* KO 1.15 strains were divided according to the pH condition and the strain in a similar way (Fig. S6). During the pH shift, a high number of DEG was observed in the KO 1.15 strain, with less than 50% shared with the WT (Figs S6, S7). To note that the Pt48498 gene, which has an unknown function, showed no reads in the *Ptnpf2* KO strain 1.15, indicating a random Cas9 off‐target mutation which was confined to the gene. The sequencing of the same gene in the other independent mutant, *Ptnpf2* KO 1.16, showed indeed no mutations (Fig. S8). While such off‐target mutation could, in principle, partly contribute to global gene expression patterns, the physiological changes observed in the mutant backgrounds appear to be independent of this unintended mutation. The consistency of the phenotype displayed by the two independent mutants strongly supports the conclusion that the observed traits are linked to the targeted *PtNPF2* gene. Additional qPCR analyses performed on the *Ptnpf2* KO strain 1.16 (Table S2) indicate a similar trend in the gene expression patterns of this strain compared with 1.15.

**Fig. 4:**
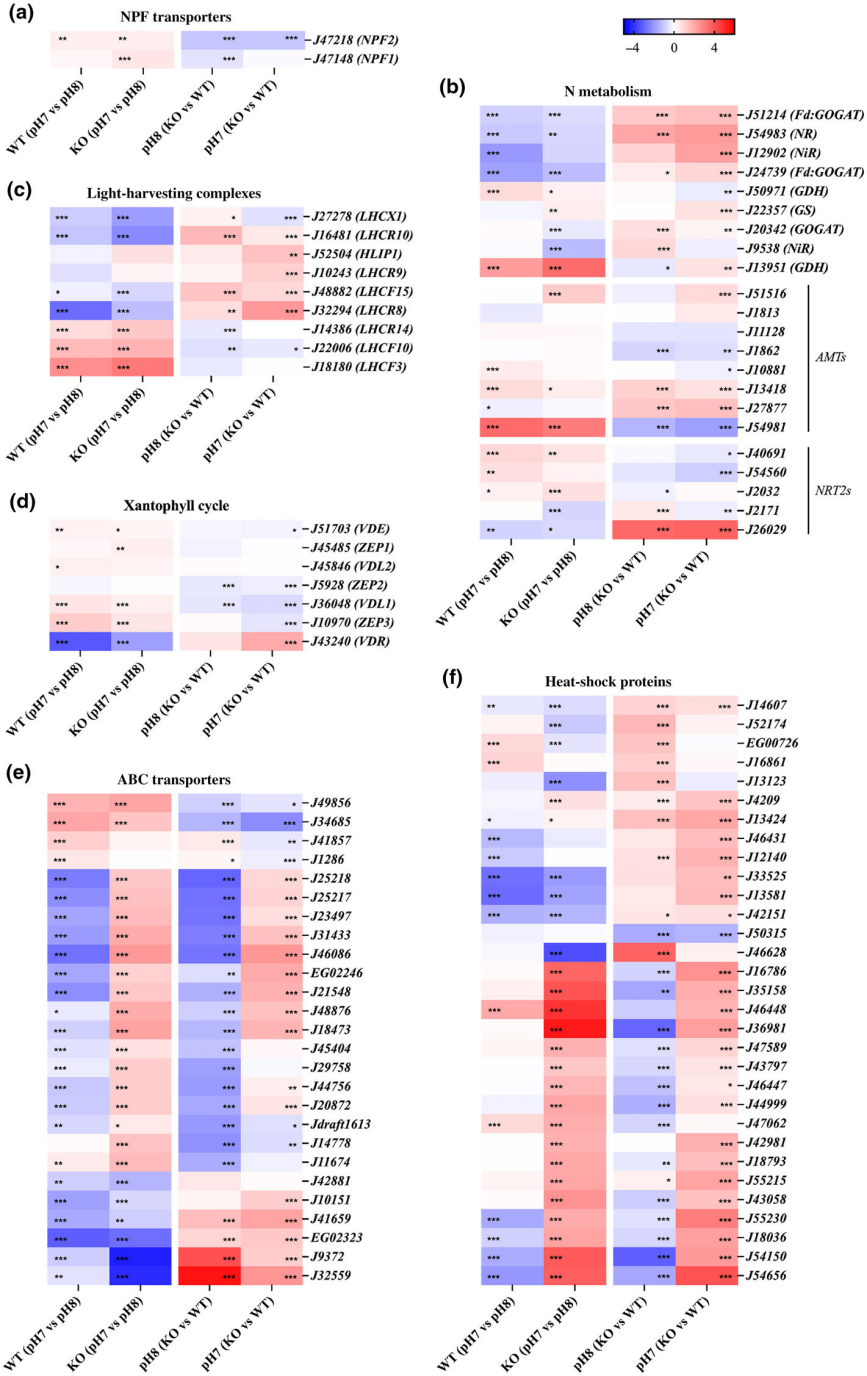
Heatmaps showing selected categories of genes regulated in *Phaeodactylum tricornutum* strains after the shift from normal to low pH. *P. tricornutum* wild‐type (WT) and *Ptnpf2* knockout (KO) mutant 1.15 after 24 h from shift from pH 8 to pH 7, performed in 882 μM NaNO_3_ as nitrogen (N) source. Heatmaps represent expression values for a set of significantly (*P* < 0.05) variable (Log_2_ fold change (LFC) > 1) genes. Comparisons show differences between different pH conditions on the same strain: WT (pH 7 vs pH 8) and WT (pH 7 vs pH 8) on the left; and differences between different strains at the same pH condition: pH 8 (KO vs WT) and pH 7 (KO vs WT) on the right. Differentially expressed genes are grouped according to the pathway: (a) target genes and *PtNPFs*, (b) N transport and N‐related genes, (c) light‐harvesting complexes, (d) genes involved in the xanthophyll cycle, (e) ATP‐binding cassette (ABC) transporters and (f) heat‐shock proteins encoding genes. Red shadows indicate upregulated genes while blue is used for downregulated genes, with the bar showing LFCs. Statistical significance: *, *P* < 0.05; **, *P* < 0.01; *** *P* < 0.001.

The expression levels of specific genes were further investigated for metabolic pathways or categories suggested to be involved in the processes affected in the mutants. In different contrasts shown in Fig. 4, the first column shows expression changes related to the response to low pH in WT cells, while the other columns show expression changes dependent on the *PtNPF2* loss of function. *PtAPT*, the selection gene, and *PtNPF2* showed a downregulation in the *Ptnpf2* KO strain, possibly due to an effect of its mutation on RNA stability (Fig. 4a). Since *PtNPF2* is a putative NO_3_^−^ transporter, N metabolism‐related genes were studied: as a general pattern, most pH‐responsive genes showed an inverse gene expression regulation in *Ptnpf2* KO strains (Fig. 4b). As examples, NO_3_^−^ and NO_2_^−^ reductases (*NR* and *NiR*), together with the Ferredoxin‐dependent glutamate synthases (*Fd:GOGAT*) and the plasma membrane localized *PtNRT2* (J26029), were generally downregulated in low pH, but their average expression levels were observed to be significantly higher in the KO strain than in the WT both in control and low pH (Fig. 4b; Table S2).

As previous photobiological analyses highlighted an impaired photosynthetic efficiency and electron transport in *Ptnpf2* KO mutants, as well as an increased NPQ (Fig. 3), the expression levels of photosynthesis associated genes were investigated. Also in these cases, pH‐responsive genes showed an opposite gene expression pattern in the *Ptnpf2* KO strain compared with WT (Fig. 4c,d). Interestingly, five LHC encoding genes, known to be involved in photo‐acclimation processes (Nymark *et al*., 2013; Valle *et al*., 2014), were shown to be significantly upregulated in the *Ptnpf2* KO strain compared with the WT, and even more induced when exposed to low pH conditions (Fig. 4c). By contrast, *PtLHCX1*, a key gene involved in NPQ processes in *P. tricornutum* (Bailleul *et al*., 2010; Buck *et al*., 2019), was slightly or not regulated in *Ptnpf2* KO strains exposed to low pH, with respect to the WT (Fig. 4c; Table S2), suggesting LHCX1 not to be a key factor driving the NPQ observed phenotype (Fig. 3d). Also, *PtLHCX2, PtLHCX3* and *PtLHCX4* (Bailleul *et al*., 2010) were observed not to be significantly regulated (Table S3).

NPQ depends on several factors, including the abundance of LHCX proteins and the de‐epoxidation state of xanthophylls, the latter being strongly influenced by the activity and quantity of enzymes involved in the xanthophyll cycle. Interestingly, an increased xanthophyll pool was observed in *Ptnpf2* KO strains exposed to low pH, which is consistent with enhanced photoprotective capacity and may contribute to the higher NPQ measured in the same pH condition. At this pH, the Dd pool was significantly higher in *Ptnpf2* KO strains, while diatoxanthin levels were below the detection threshold and β‐carotene showed no significant difference (Figs 3e, S5). These results suggest that PtNPF2 influences how pH modulates the activity or abundance of enzymes involved in xanthophyll biosynthesis. At the transcriptional level, although the gene encoding VDE did not show major expression variations, the VDE‐related genes *PtVDR* and *PtVDL1* exhibited expression profiles following NPQ profile. Specifically, *PtVDR* was upregulated and *PtVDL1* downregulated in *Ptnpf2* KO strains compared with the WT strain, but only at pH 7 (Figs 3e, 4d). Also, *PtVDR* showed strain‐specific variation between the two *Ptnpf2* KO lines (Table S2). VDL1 is known to catalyse the conversion of violaxanthin (Vx)/zeaxanthin (Zx) to Dd/diatoxanthin (Dautermann *et al*., 2020; Li *et al*., 2024), while no specific function has yet been assigned to VDR (Wei *et al*., 2024). Although the carotenoid biosynthesis pathway in diatoms still has many unresolved steps, these results collectively suggest that PtNPF2 influences, directly or indirectly, the regulation of the xanthophyll biosynthesis pathway in response to a decrease in pH, thereby modulating NPQ capacity.

Among other gene categories standing out in the metabolic remodelling that occurred in response to *PtNPF2* KO were ATP‐binding cassette (ABC) transporters, a superfamily of transmembrane proteins critical in maintaining redox homeostasis (Wilkens, 2015; Kou *et al*., 2020). They represent an interesting category of pH‐responsive genes, since most of them were observed to be downregulated in response to low pH in the WT, but reverting this pattern into upregulation in the *Ptnpf2* KO exposed to low pH (Fig. 4e; Table S2). Also, significant gene regulation was observed in heat‐shock protein (HSP)‐related genes (Fig. 4f), known to be involved in redox balance and internal homeostasis control in diatoms (Dong *et al*., 2016), most of which displayed a significant upregulation in KO strain, especially in low pH conditions (Fig. 4f; Table S2).

## Discussion

Intracellular ion balance is an essential parameter to support optimal metabolic activity and to face environmental fluctuations. It could also be an important trait in defining fitness and ecological success of many organisms. This equilibrium is finely tuned by a regulation network which involves channels and transporters located on different cellular membranes, allowing exchanges with the external environment but also between different intracellular compartments and organelles. However, due to the complexity of multiple simultaneous processes, the mechanisms and molecular actors involved in such regulations often remain poorly known, especially in less studied organisms like secondary endosymbiotic microalgae.

**Fig. 5:**
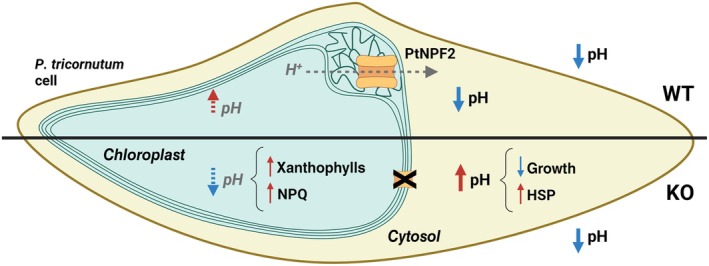
Schematic representation of a *Phaeodactylum tricornutum* cell illustrating physiological and transcriptional changes occurring in *Ptnpf2* knockout (KO) strains exposed to low pH. The upper part of the *P. tricornutum* cell represents a wild‐type (WT) cell, whereas the lower part represents a *Ptnpf2* KO cell. Arrows indicate the change direction, with red arrows denoting induction or increase and blue ones indicating inhibition or reduction. Solid arrows with black labels represent experimentally validated data, indicating confirmed changes in cell metabolism or physiology, whereas dotted arrows with grey italic labels denote hypothesized changes inferred by present data but still requiring direct experimental validation. PtNPF2 is depicted as an orange transporter located in the periplastidial compartment (PPC), between two of the four membranes of the diatom chloroplast. Structural elements are simplified; the drawing is not in scale. H^+^, protons; HSP, genes encoding for heat‐shock proteins; NPQ, nonphotochemical quenching. This figure was created in BioRender (https://BioRender.com/0ru4kz4).

This work highlighted metabolic alterations of *P. tricornutum* WT cells, which supported a fine regulation of the intracellular ion equilibrium in response to the shift to low pH. Cell response to low environmental pH is an important but still poorly understood topic: many recent researches focused on climate changes investigated the effects of high CO_2_ on diatom cells (Huang *et al*., 2019; Zhang *et al*., 2020; Jin *et al*., 2022), but increased CO_2_ into seawater also means decreased pH (Caldeira & Wickett, 2005; Fabry *et al*., 2008), and often the two parameters have a different effect on cell physiology. For example, high CO_2_ often results in more efficient photosynthesis, while low pH often unbalances internal homeostasis (Huang *et al*., 2019; Shi *et al*., 2019). Also, scientists examined the effects of decreased pH on growth and biodiversity (DeNicola, 2000; Taraldsvik & Myklestad, 2000; Gao *et al*., 2020; Shang *et al*., 2024), but few transcriptomic data have been recorded in acidified conditions (Li *et al*., 2023). In this work, low pH was observed not to affect growth or physiological photosynthetic parameters in the first 48 h investigated (Figs 2, 3, 5, S9a). However, cells lowered their pH_i_ to maintain the cross‐membrane electrochemical gradient for H^+^ efflux, confirming what was already observed in previous works (Shi *et al*., 2019). Many genes involved in photosynthetic processes were shown to be regulated in response to the low pH exposure in *P. tricornutum* WT, as well as key genes involved in N assimilation, the latter particularly downregulated (Fig. 4b). These were defined as pH‐responsive genes. Furthermore, ABC transporters were observed to be the family showing the most widespread regulation, with one third of genes differentially expressed, suggesting once again to be the most involved in ion balance homeostasis in response to pH changes (Figs 4, 5, S9a). This represents the first attempt of *P. tricornutum* cells to overcome intracellular ion imbalance caused by environmental pH change.

In this work, PtNPF2, a member of a putative N transporters family, has been chosen to study pH_i_ balance. PtNPF2, in fact, is phylogenetically and structurally close to bacteria POTs and is known to be regulated by environmental pH, suggesting a link with pH‐related processes (Santin *et al*., 2021).

Strains expressing PtNPF2 in frame with two different fluorescent tags showed that PtNPF2 is localized in a small compartment close to the nucleus and between the two chloroplast lobes (Fig. 1), corresponding to the PPC (Maier *et al*., 2015, 2022; Moog *et al*., 2019; Marter *et al*., 2020). PPC is a compartment located between the membranes of the diatom chloroplast, and it is surrounded by the periplastidial membrane (PPM). Both PPC and PPM have been proposed to have key roles in regulating the fluxes of molecules and ions between cytosol and the four membranes of the chloroplast, acting as a communication link between cellular metabolism and photosynthetic activity (Tachibana *et al*., 2011; Maier *et al*., 2022). The localization of the PtNPF2, which is a transmembrane protein, into the PPC suggests that it may be part of a vesicular network involved in the transport of different molecules from the cytosol to the chloroplast through the PPC compartment (Gibbs, 1979; Flori *et al*., 2016).

The first indication of the PtNPF2 role in ion and pH balance was given by its significant upregulation at low pH, which together with the presence of the ExxER structural motif responsible for H^+^‐coupled bond, suggested a possible role of PtNPF2 in H^+^‐transport and pH regulation (Santin *et al*., 2021). While no physiological differences were observed in *Ptnpf2* KO mutants and WT strains exposed to normal pH, the transcriptomic analysis indicated that a metabolic remodelling occurred in the cell, probably acting in order to overcome PtNPF2 absence (Figs 4, S6, S9b). Key photosynthesis‐related genes, together with the N assimilation pathway, indeed were observed to be upregulated in the *Ptnpf2* KO mutants under standard pH levels (Fig. 4).

These hints are further supported by the *Ptnpf2* KO phenotype after the shift to low pH. In fact, the KO of this gene caused a growth lag at low pH, associated with a photosynthetic phenotype. Two transient responses were observed in the first days after the shift to low pH, including a reduced growth in KO strains and an impaired electron transport rate (Fig. 2). As symptoms of an overall stress response, these physiological data were confirmed by gene expression results, highlighting a high number of genes involved in oxidation–reduction processes as well as heat‐shock response (Figs 4, 5, S9c) strongly regulated in the mutant after the shift to low pH. Moreover, ABC transporters, a big family of transporters known to be critical in maintaining redox homeostasis (Wilkens, 2015; Kou *et al*., 2020), were shown to be strongly upregulated in the *Ptnpf2* KO strain, supporting the hypothesis of its role in maintaining intracellular ion homeostasis by regulating ion transport across membranes.

These short‐term differences were then complemented by steady‐state responses, such as the lower maximal yield of dark‐adapted PSII (*F*_v_/*F*_m_) observed in *Ptnpf2* KO mutants after the shift to low pH (Fig. 3). This suggests that the reduced photosynthetic efficiency and transport rate could be partially due to structural reorganization of PSII after the low pH shift. Interestingly, the *Ptnpf2* KO strains also showed a higher maximum NPQ in low pH, compared with both WT and OE strains (Figs 3, 5). Mechanisms activating NPQ are different from those involved in PSII efficiency and electron transport, and included as follows: (1) direct enzymatic control, by which NPQ dynamics over a light curve or relaxation kinetics in the dark is proportional to the net de‐epoxidation rate of the xanthophyll cycle (Blommaert *et al*., 2021), with the main enzymes regulators being luminal pH and NADPH in the stroma (Goss & Jakob, 2010), (2) long‐term acclimation processes, which determine alterations in the gene expression or changes in the activity of xanthophyll‐cycle pigments and enzymes, and (3) LHCX proteins, which define the NPQ potential (Buck *et al*., 2019; Croteau *et al*., 2024). The last two regulatory mechanisms described above are corroborated by our experimental data.

In fact, although no transcriptional regulation of *VDE* was detected, its substrate, Dd, accumulates more in *Ptnpf2* KO strains than in the WT when exposed to low pH (Figs 4, S5). Transcriptomic analyses also revealed differences between the *Ptnpf2* KO strains and the WT strain at low pH only: *PtVDL1* and *PtZEPs* were downregulated, while *PtVDR* was significantly upregulated in the KO strains. These results indicate that PtNPF2 likely influences the regulation of xanthophyll biosynthesis, thus affecting photoprotective capacity and contributing to the increase in NPQ observed in *Ptnpf2* KO mutants under low pH conditions. To note that xanthophyll‐cycle pigments (Vx and Zx) can directly contribute to NPQ by occupying shared binding sites in the light‐harvesting antennae, even in species that normally rely on the Dd cycle (Giossi *et al*., 2025). Because *de novo* xanthophyll synthesis depends on *ZEP2* activity and on the size and accessibility of the Vx‐Zx precursor pool, which may include a periplastidial localization, PPC localization of PtNPF2 and its role in pH modulation could directly affect enzyme activity and substrate availability. Together, the transcriptional changes and altered periplastidial environment provide a coherent explanation for the higher xanthophyll accumulation and enhanced NPQ observed in *Ptnpf2* KO strains under low pH, linking the PtNPF2 mutation to both gene expression and local physicochemical regulation of photoprotection.

In this context, the higher xanthophyll content and transcriptional modifications detected in low pH, dark‐adapted *Ptnpf2* KO strains suggest that these cells adopt a constitutive acclimatization state in which xanthophyll biosynthesis and photoprotection pathways are differentially activated. Such a state can induce chronic protein or membrane stress, potentially affecting the chloroplast redox or pH‐sensing mechanisms that normally regulate NPQ induction. Therefore, the PtNPF2 mutation appears to steer cells towards a ‘stress response’ phenotype, enabling them to tolerate an acidified environment through an energetically costly metabolic reorganization.

Also, while in WT *P. tricornutum*, as well as other diatoms, cytosolic pH_i_ trend followed extracellular pH values, so that pH_i_ decreases as environmental pH decreases (Shi *et al*., 2019; Figs 3, 5, S9a), *Ptnpf2* KO mutants showed a higher relative pH_i_ when exposed to low pH (Figs 3, 5, S9c), with values very close to those measured at normal pH. This is particularly interesting as very few studies have investigated physiological remodelling in response to pH changes. Generally, microalgae adjust their pH_i_ in response to the external one by using different strategies: activating energy‐dependent mechanisms of H^+^ efflux, such as H^+^ transporters (Taylor *et al*., 2012; Shi *et al*., 2019), or allowing H^+^ channels to function for passive H^+^ efflux. Consequently, the unchanged pH_i_ in *Ptnpf2* KO mutants after the pH shift could be explained by a mechanism controlling H^+^ transport, linked to PtNPF2, in turn impairing H^+^ transport across intracellular compartments. One possible hypothesis could be that PtNPF2, localized in the PPC, is involved in driving H^+^ from the chloroplast stroma to the cytosol (Fig. 5). Disruption of this transport could consequently impair cytosolic pH regulation under low external pH conditions, when tight control is essential, while simultaneously affecting chloroplast internal homeostasis. This dual alteration might compromise photosynthetic processes, which are highly sensitive to pH changes (Fig. 5).

Although all the data strongly support the hypothesis that PtNPF2 could function as a H^+^ transporter, our preliminary analysis using the heterologous *Xenopus* expression system did not reveal transport activity for either NO_3_^−^ or dipeptides as substrates, across a range of pH levels. Consequently, we were unable to experimentally confirm that our protein functions as a NO_3_^−^/H^+^‐coupled transporter under the tested conditions (Methods S2; Fig. S10). The *Xenopus laevis* oocytes provide a powerful tool for the expression and characterization of plant membrane proteins (Miller & Zhou, 2000; Léran *et al*., 2020). However, for diatoms, only a few examples of heterologous expression of transporters have been reported to date (Hildebrand *et al*., 1998; Knight *et al*., 2016; Santin *et al*., 2024). Further experimentation will be necessary to advance this line of research.

Overall, these data highlight how a decrease in external pH can stimulate a response in the cytosolic pH homeostasis (Fig. 3), which in turn can be reflected in molecular and biochemical mechanisms acting inside chloroplasts (Figs 3, 4, 5). This complicated cross‐talk between different compartments is still not deeply investigated, as it involves many mechanisms and key players present in different compartments of the cell. One of these key players could be represented by PtNPF2, which, acting as a putative H^+^ transporter between chloroplast membranes, would be involved in maintaining the communication between the chloroplast and the rest of the cell, thus partially regulating photosynthetic responses to external stimuli.

The transcriptomic data obtained in this work represent a novel valuable dataset, contributing information on the diatom responses to pH changes (Table S3). To enhance discoverability and usability, the gene expression data will be uploaded to the DiatOmicsBase database (https://www.diatomicsbase.bio.ens.psl.eu/), with free and easy access to the scientific community.

As a result of this study, new insights on PtNPF2 functional role have been provided and new hypotheses have been proposed: PtNPF2 has been shown to localize in the PPC and to be probably involved in H^+^ transport across chloroplast membranes, thus partially controlling pH and ion intracellular homeostasis. This puts PtNPF2 in a crucial position in assuring the continuity of gradients between intracellular compartments of the diatom cell. Moreover, in a context of ongoing climate changes, where ocean acidification represents one of the major threats to understand, this work provides new information on diatoms' possible strategies to face pH changes.

The transient phenotypes observed in both *Ptnpf1* (Santin *et al*., 2024) and *Ptnpf2* KO mutants suggest that both members of this family have evolved to play critical roles in fine‐tuning complex and highly regulated mechanisms which are required at the interface with the external environment for a correct intracellular distribution of molecules and resources (Santin *et al*., 2024). Each component contributes to enabling diatoms to perceive and respond effectively to unpredictable intra‐ and extracellular changes. Furthermore, these findings demonstrate that both family members have evolved to participate in specific cellular processes, including ion balance and communication between different cell compartments in response to environmental changes, particularly in relation to pH stress.

## Competing interests

None declared.

## Author contributions

AS, AF, MIF and Alessandra Rogato designed the research. AS, DC, RS, Antonella Ruggiero, SRS, LMdlR, CC‐F, SB, BB and Alessandra Rogato performed the research. BL, AF, Alessandra Rogato and MIF contributed new reagents or analytical tools. AS, MTR, DC, RS, BL, BB and Alessandra Rogato analysed the data. AS and Alessandra Rogato wrote the first draft of paper. AS, MTR, MC, MRd'A, BB, AF, MIF and Alessandra Rogato reviewed the paper.

## Disclaimer

The New Phytologist Foundation remains neutral with regard to jurisdictional claims in maps and in any institutional affiliations.

## Supporting information


**Fig. S1** Screening of *Phaeodactylum tricornutum Ptnpf2* knockout (KO) mutants.
**Fig. S2** Subcellular localization of PtNPF2 in *Phaeodactylum tricornutum* through PtNPF2‐YFP and GFP‐PtNPF2 fusion protein expression, compared with wild‐type (WT).
**Fig. S3** Subcellular co‐localization of PtNPF2 and periplastidial compartment (PPC) in *Phaeodactylum tricornutum*.
**Fig. S4** Growth curves of *Phaeodactylum tricornutum* wild‐type, PtNPF2‐YFP overexpressing and knockout strains.
**Fig. S5** Pigment composition of *Phaeodactylum tricornutum* strains following pH shift.
**Fig. S6** Overview of the transcriptome of *Phaeodactylum tricornutum* strains after the shift from normal to low pH.
**Fig. S7** Gene expression differences between *Phaeodactylum tricornutum* strains after the shift from normal to low pH.
**Fig. S8** Sequencing of the off‐target gene Pt48498 on the *Ptnpf2* knockout (KO) strain 1.16.
**Fig. S9** Representation of physiological and transcriptional changes in *Phaeodactylum tricornutum* wild‐type (WT) and *Ptnpf2* knockout (KO) strains exposed to normal and low pH.
**Fig. S10** Analysis of nitrate and dipeptide uptake by PtNPF2 in *Xenopus laevis* oocytes.
**Methods S1** Growth experiments.
**Methods S2** Heterologous expression in *Xenopus laevis* oocyte.
**Table S1** List of oligonucleotides information.
**Table S2** qPCRs performed on selected genes on *Phaeodactylum tricornutum* wild‐type (WT) and *Ptnpf2* knockout (KO) strains 1.15 and 1.16.
**Table S3** Complete overview of the entire transcriptome of *Phaeodactylum tricornutum* wild‐type (WT) and *Ptnpf2* knockout (KO) strain 1.15.
Please note: Wiley is not responsible for the content or functionality of any Supporting Information supplied by the authors. Any queries (other than missing material) should be directed to the *New Phytologist* Central Office.

## Data Availability

The authors confirm that the data supporting the findings of this study are available within the article and its supplementary materials, specifically in Fig. S4; Tables S2 and S3. The raw sequencing data and assembly used in this study have been deposited in ENA under the following accession ID: E‐MTAB‐14821.
