# Supplementary material for: NPF2 is involved in intracellular pH regulation and ion balance in the diatom Phaeodactylum tricornutum
Source: New Phytol. 2026 Apr 21;250(6):3885–99. doi: 10.1111/nph.71169 (PMC13193496; doi:10.1111/nph.71169)
Supplement: Supplementary file 1 — Fig. S1 Screening of Phaeodactylum tricornutum Ptnpf2 knockout (KO) mutants. Fig. S2 Subcellular localization of PtNPF2 in Phaeodactylum tricornutum through PtNPF2‐YFP and GFP‐PtNPF2 fusion protein expression, compared with wild‐type (WT). Fig. S3 Subcellular co‐localization of PtNPF2 and periplastidial compartment (PPC) in Phaeodactylum tricornutum. Fig. S4 Growth curves of Phaeodactylum tricornutum wild‐type, PtNPF2‐YFP overexpressing and knockout strains. Fig. S5 Pigment composition of Phaeodactylum tricornutum strains following pH shift. Fig. S6 Overview of the transcriptome of Phaeodactylum tricornutum strains after the shift from normal to low pH. Fig. S7 Gene expression differences between Phaeodactylum tricornutum strains after the shift from normal to low pH. Fig. S8 Sequencing of the off‐target gene Pt48498 on the Ptnpf2 knockout (KO) strain 1.16. Fig. S9 Representation of physiological and transcriptional changes in Phaeodactylum tricornutum wild‐type (WT) and Ptnpf2 knockout (KO) strains exposed to normal and low pH. Fig. S10 Analysis of nitrate and dipeptide uptake by PtNPF2 in Xenopus laevis oocytes. Methods S1 Growth experiments. Methods S2 Heterologous expression in Xenopus laevis oocyte. Table S1 List of oligonucleotides information. Table S2 qPCRs performed on selected genes on Phaeodactylum tricornutum wild‐type (WT) and Ptnpf2 knockout (KO) strains 1.15 and 1.16. Table S3 Complete overview of the entire transcriptome of Phaeodactylum tricornutum wild‐type (WT) and Ptnpf2 knockout (KO) strain 1.15. Please note: Wiley is not responsible for the content or functionality of any Supporting Information supplied by the authors. Any queries (other than missing material) should be directed to the New Phytologist Central Office. [file NPH-250-3885-s001.pdf]

***New Phytologist* Supporting Information**

Article title: NPF2 is involved in intracellular pH regulation and ion balance in the diatom *Phaeodactylum tricornutum*

Authors: Anna Santin, Monia Teresa Russo, Dany Croteau, Antonella Ruggiero, Sara Russo Spena, Laura Morales de los Rios, Claire Corratge-Faillie, Seleem Brignone, Benoit Lacombe, Remo Sanges, Maurizio Chiurazzi, Maurizio Ribera d'Alcalà, Benjamin Bailleul, Angela Falciatore, Maria Immacolata Ferrante, Alessandra Rogato

Article acceptance date: 17 March 2026

The following Supporting Information is available for this article:

Supporting Figures.

Fig S1. Screening of *P. tricornutum Ptnpf2* knock-out (KO) mutants. A) Agarose gels showing the PCR screening of the *PtAPT* and *PtNPF2* genes on *P. tricornutum* wild-type (WT) and transformed cells positive to selection, M represents the molecular marker. B) *P. tricornutum* wild-type and *Ptnpf2* KO mutants schemes and chromatograms showing Cas9 different effects on the two KO alleles: *P. tricornutum* wild-type sequence shows the structural model of PtNPF2 wild-type and gRNAs and primer couples used for sequencing; *Ptnpf2* KO mutants 1.15 and 1.16 mutation scheme and comparison between wild-type and KO chromatograms showing insertion as grey bars and deletions as grey dotted lines on both alleles.

Fig S2. Subcellular localization of PtNPF2 in *P. tricornutum* through PtNPF2-YFP and GFP-PtNPF2 fusion protein expression, compared to wild-type (WT). From left to right: bright-field (BF) images in grey, chlorophyll *a* fluorescence in magenta, YFP fluorescence in green, GFP fluorescence in cyano, HOECHST-stained nuclei in blue and the merged image. Scale bar: 5 µm.

Fig S3. Subcellular co-localization of PtNPF2 and periplastidial compartment (PPC) in *P. tricornutum*. Co-localization analysis was performed using the PtNPF2-YFP fusion protein expressed in the overexpression (OE) 2 strain (background and control) and the BTS-Hsp70-mRuby fusion protein, which is known to localize to the PPC. Images from left to right show: bright-field (BF) in grey, chlorophyll *a* fluorescence in magenta, YFP fluorescence in green, mRuby fluorescence in orange, HOECHST-stained nuclei in blue, and the merged image. Scale bar: 5 µm.

Fig S4. Growth curves of *P. tricornutum* wild-type, PtNPF2-YFP overexpressing, and knock-out strains. Growth curves of *P. tricornutum* wild-type (WT), two PtNPF2-YFP overexpressing (OE) strains, two *Ptnpf2* knock-out (KO) mutants and an additional *Ptnpf1* KO mutant, in different NO_3^- concentrations, N sources and pH conditions: A) pH 8 - 882 µM $NaNO_3$ (control pH and NO_3 concentration present in standard F/2 medium); B) pH 8 - 50 µM $NaNO_3$ as N starvation condition; C) pH 8 - 882 µM NH_4Cl and D) pH 8 - 882 µM urea as alternative N sources; E) pH 9 - 882 µM $NaNO_3$, F) pH 7 - 882 µM $NaNO_3$ and G) pH 6 - 882 µM $NaNO_3$ as different pH conditions; H) pH 7 - 882 µM NH_4Cl and I) pH 7 - 882 µM urea as combined low pH and alternative N sources; L) pH 8 - 882 µM KNO_3 as alternative combined salt in NO_3 compound. *In vivo* chlorophyll *a* fluorescence was used for measurements as proxy for growth. Error bars represent standard deviations (+/- SD) of three biological replicates. Statistical significance: * indicates $p < 0.05$; ** for $p < 0.01$ and * for $p < 0.001$.

Fig S5. Pigment composition of *P. tricornutum* strains following pH shift. Pigment analysis performed on *P. tricornutum* strains, wild-type (WT) and *Ptnpf2* knock-out (KO) mutants 1.15 and 1.16, after 24 hours from pH shift. Chlorophyll *c*, β-carotene, Diadinoxanthin and Fucoxantin molar content, normalized on chlorophyll *a* molar content. Error bars represent standard deviations (+/- SD) of three biological replicates. Statistical significance: * indicates $p < 0.05$; ** for $p < 0.01$ and * for $p < 0.001$.

Fig S6. Overview of the transcriptome of *P. tricornutum* strains after the shift from normal to low pH. *P. tricornutum* wild-type (WT) and *Ptnpf2* knock-out (KO) mutant 1.15 after 24 hours from shift from pH 8 to pH 7, performed in 882 µM $NaNO_3$. A) Principal component analysis (PCA) shows the effect of strain and pH on the two axes. B) Numbers of differentially expressed genes (DEGs) for all comparisons. In the "KO vs WT" comparison “WT” was set as reference, while in "pH7 vs pH8" comparison “pH8” was set as reference. C) Venn diagrams and clustering analysis of transcriptomic results, combining single comparisons to find strain specific genes regulated in low pH. D) Total functional enrichment analysis of DEGs in *P. tricornutum*: Y-axis

indicates enriched pathways, upper X-axis represents the enrichment score, represented by dots, while lower X-axis displays the number of DEGs, shown as bars.

Fig. S7. Gene expression differences between *P. tricornutum* strains after the shift from normal to low pH. *P. tricornutum* wild-type (WT) and *Ptnpf2* knock-out (KO) mutant 1.15 after 24 hours from shift from pH 8 to pH 7, performed in 882 μM $NaNO_3$. Heatmap of the top 40 differentially expressed genes (DEGs) in the *P. tricornutum* transcriptome. The intensity of the orange/purple colours represents the normalized expression level. The rows have been sorted according to the gene clustering tree following Pearson correlation. The left-hand bar is colour-coded according to the functions of the genes.

Fig. S8. Sequencing of the off-target gene Pt48498 on the *Ptnpf2* knock-out (KO) strain 1.16. As an off-target gene was found to be mutated in *Ptnpf2* KO strain 1.15, the same gene was sequenced in the *Ptnpf2* KO strain 1.16 genome, to exclude that this off-target could be responsible of the observed growth and photosynthetic phenotype shared by the two mutants. No mutations were found for gene Pt48498 in strain 1.16. A) PCRs on gDNA, B) PCRs on cDNA and C) sequencing of different wild-type and *Ptnpf2* knock-out strains.

Fig. S9. Representation of physiological and transcriptional changes in *P. tricornutum* wild-type (WT) and *Ptnpf2* knock-out (KO) strains exposed to normal and low pH. A) Changes occurring during pH acidification in wild-type cells, meaning in cells shifted from pH 8 to pH 7. B) Changes occurring in the *Ptnpf2* KO mutant compared to the wild-type, in normal pH (pH 8). C) Changes occurring in the *Ptnpf2* KO mutant compared to the wild-type, after the shift to low pH. Different colours indicate different intracellular compartments, in particular cytosol and chloroplast. PtNPF2 is represented as transporter putatively located on the periplastidial compartment (PPC), between chloroplast membranes, when present in the wild-type cells. Normal font is used for physiological parameters, italics for the transcriptional differences. Arrows indicate the change direction, with red arrows for induction or upregulation and blue ones for inhibition/reduction or downregulation. PSII eff: PSII photosynthetic efficiency; NPQ: Non-Photochemical Quenching; *LHCs*: Light-harvesting related genes; *Xant*: xanthophyll cycle related genes; *ABCs*: genes encoding for ATP-binding cassettes; *HSPs*: genes encoding for Heat-shock proteins. This figure was created in BioRender (https://BioRender.com/m9jv1yc).

Fig. S10. Analysis of nitrate and dipeptide uptake by PtNPF2 in *Xenopus laevis* oocytes. A) Non injected, AtNPF6.3 (positive control) or PtNPF2-expressing oocytes were incubated in bathing

medium containing 30 mM ^{15}N-labeled nitrate at different pH to search for ^{15}N-nitrate accumulation. B) Non injected, AtNPF8.1 (positive control) or PtNPF2-expressing oocytes were incubated in bathing medium containing 10 mM ^{13}C-labeled LEU-LEU at different pH to search for ^{13}C-LEU-LEU accumulation. Data are mean +/- SD (n = 5-10 oocytes). Statistical significance: ** indicates $p < 0.01$ and *** for $p < 0.001$. C) AtNPF8.1 (positive control) and PtNPF2 current-voltage relationships (I-V curves) in 10 mM ALA-ALA and LEU-LEU at different pH. Mean value +/-SD in n=3-5 oocytes.

Supporting Tables.

Table S1. List of oligonucleotides information. crRNAs designed for CRISPR/Cas9 proteolistic transformation (with PAM sequence underlined), primers used for plasmid construction, primers used for overexpressing and knock-out mutants screening, and primers used for qPCRs.

Table S2. qPCRs performed on selected genes on *P. tricornutum* wild-type (WT) and *Ptnpf2* knock-out (KO) strains 1.15 and 1.16. Data represent Log_2 fold-changes (± SD, three biological replicates analysed) of the comparisons described: WT (pH7 vs pH8) indicates the conditions that determined pH-responsive genes, pH8 (KO vs WT) and pH8 (KO vs WT) indicate comparison between KO and wild-type grown at different pH for 24 hours. Considering the different sensitivity of RNA-seq and qPCR, data from the two different techniques are generally in accordance.

Table S3. Complete overview of the entire transcriptome of *P. tricornutum* wild-type (WT) and *Ptnpf2* knock-out (KO) strain 1.15. Transcriptomic analysis performed before (pH 8) and after 24 hours from the pH shift (pH 7). Information includes gene IDs, annotations, KEGG functions, Log_2 Fold-Changes (LFCs) and p values adjusted for all genes regulated in the comparisons WT (pH7 vs pH8), KO (pH7 vs pH8) as well as in the comparisons pH8 (KO vs WT) and pH7 (KO vs WT). FLCs above ±1 and p values adjusted < 0.05 were considered significant.

Supporting Methods.

Methods S1. Growth experiments.

Methods S2. Heterologous expression in *Xenopus laevis* oocyte.

Supporting Figures.

Fig S1. Screening of *P. tricornutum Ptnpf2* knock-out (KO) mutants. A) Agarose gels showing the PCR screening of the *PtAPT* and *PtNPF2* genes on *P. tricornutum* wild-type (WT) and transformed cells positive to selection, M represents the molecular marker. B) *P. tricornutum* wild-type and *Ptnpf2* KO mutants schemes and chromatograms showing Cas9 different effects on the two KO alleles: *P. tricornutum* wild-type sequence shows the structural model of PtNPF2 wild-type and gRNAs and primer couples used for sequencing; *Ptnpf2* KO mutants 1.15 and 1.16 mutation scheme and comparison between wild-type and KO chromatograms showing insertion as grey bars and deletions as grey dotted lines on both alleles.

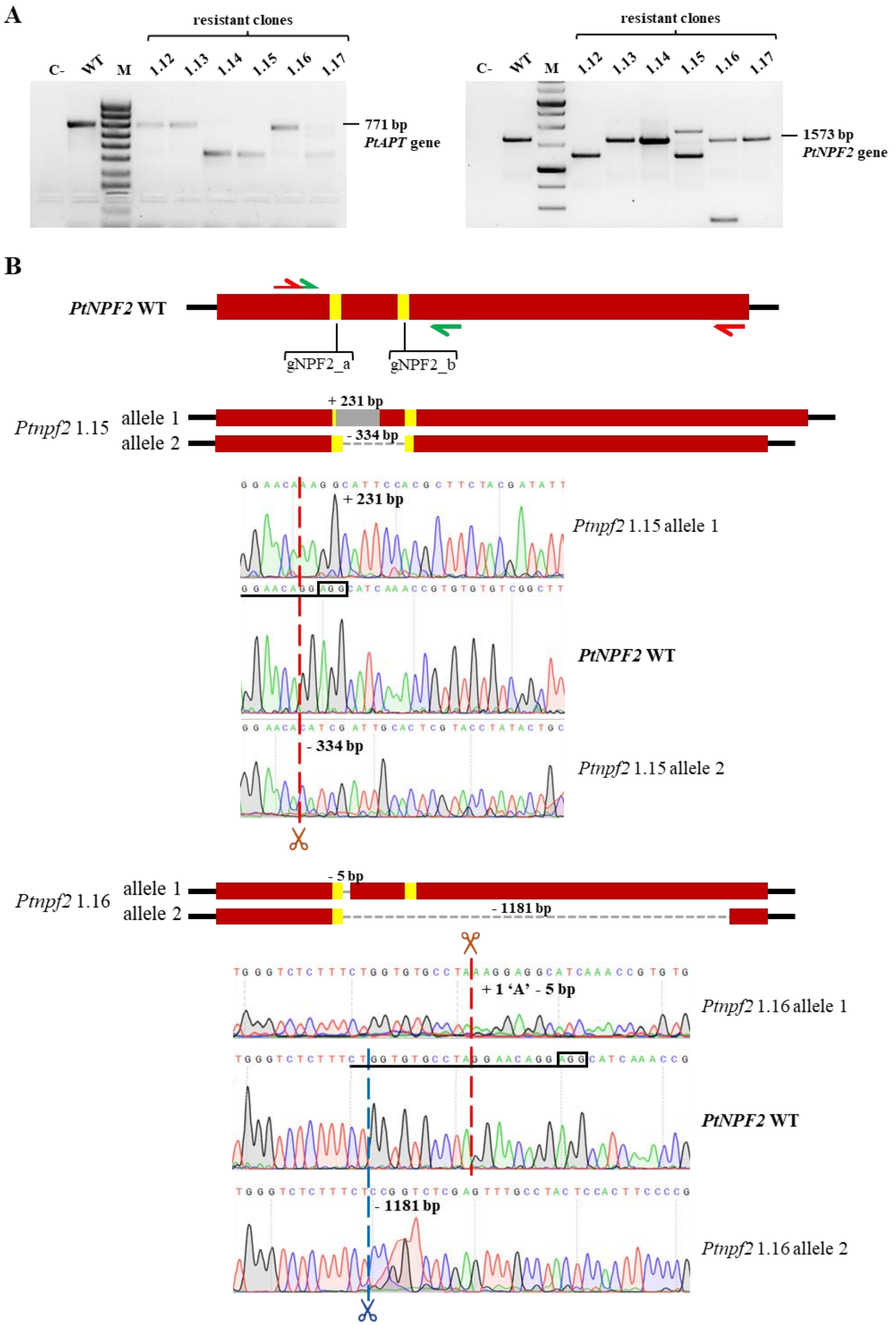

Fig S2. Subcellular localization of PtNPF2 in *P. tricornutum* through PtNPF2-YFP and GFP-PtNPF2 fusion protein expression, compared to wild-type (WT). From left to right: bright-field (BF) images in grey, chlorophyll *a* fluorescence in magenta, YFP fluorescence in green, GFP fluorescence in cyano, HOECHST-stained nuclei in blue and the merged image. Scale bar: 5 µm.

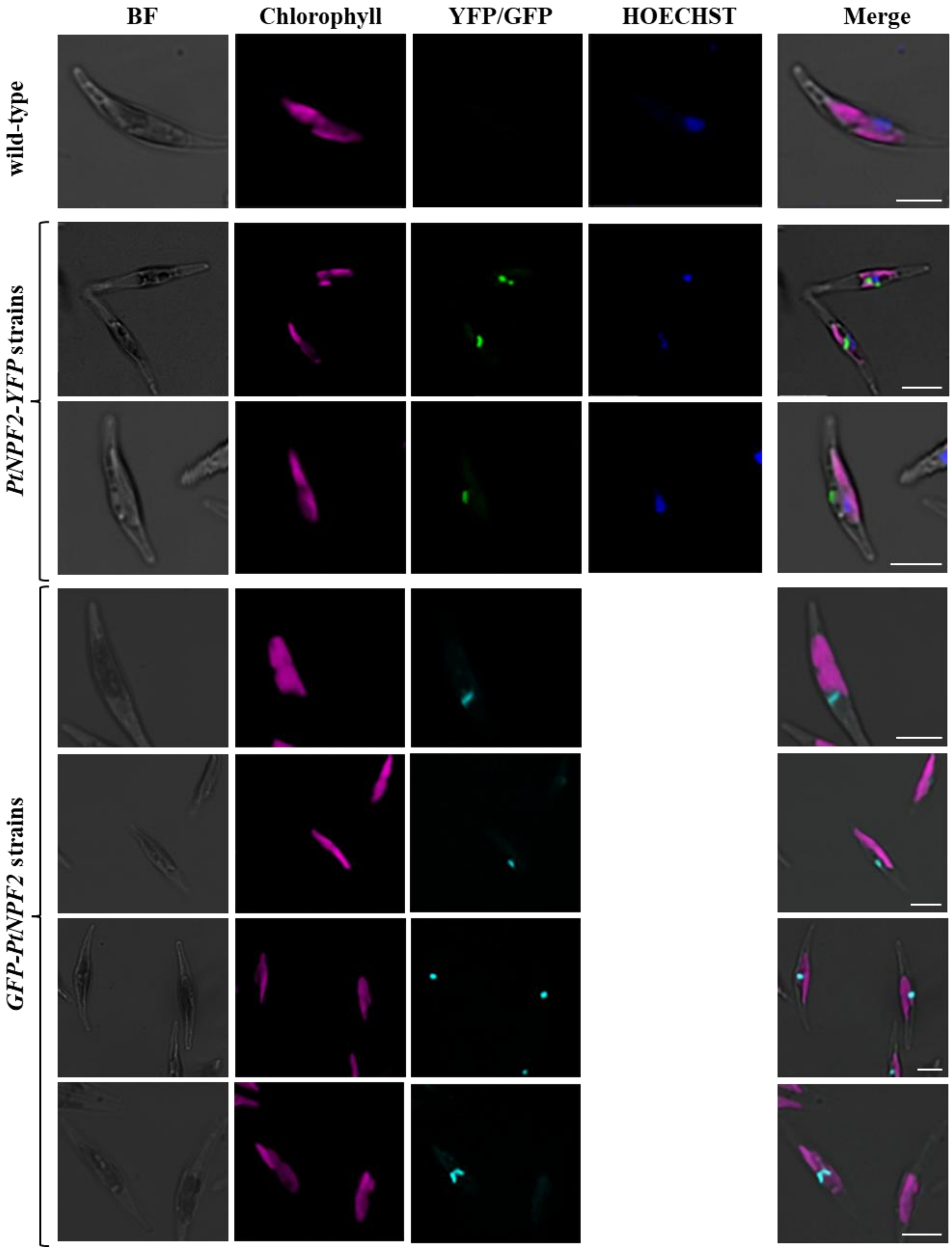

Fig S3. Subcellular co-localization of PtNPF2 and periplastidial compartment (PPC) in *P. tricornutum*. Co-localization analysis was performed using the PtNPF2-YFP fusion protein expressed in the overexpression (OE) 2 strain (background and control) and the BTS-Hsp70-mRuby fusion protein, which is known to localize to the PPC. Images from left to right show: bright-field (BF) in grey, chlorophyll *a* fluorescence in magenta, YFP fluorescence in green, mRuby fluorescence in orange, HOECHST-stained nuclei in blue, and the merged image. Scale bar: 5 μm.

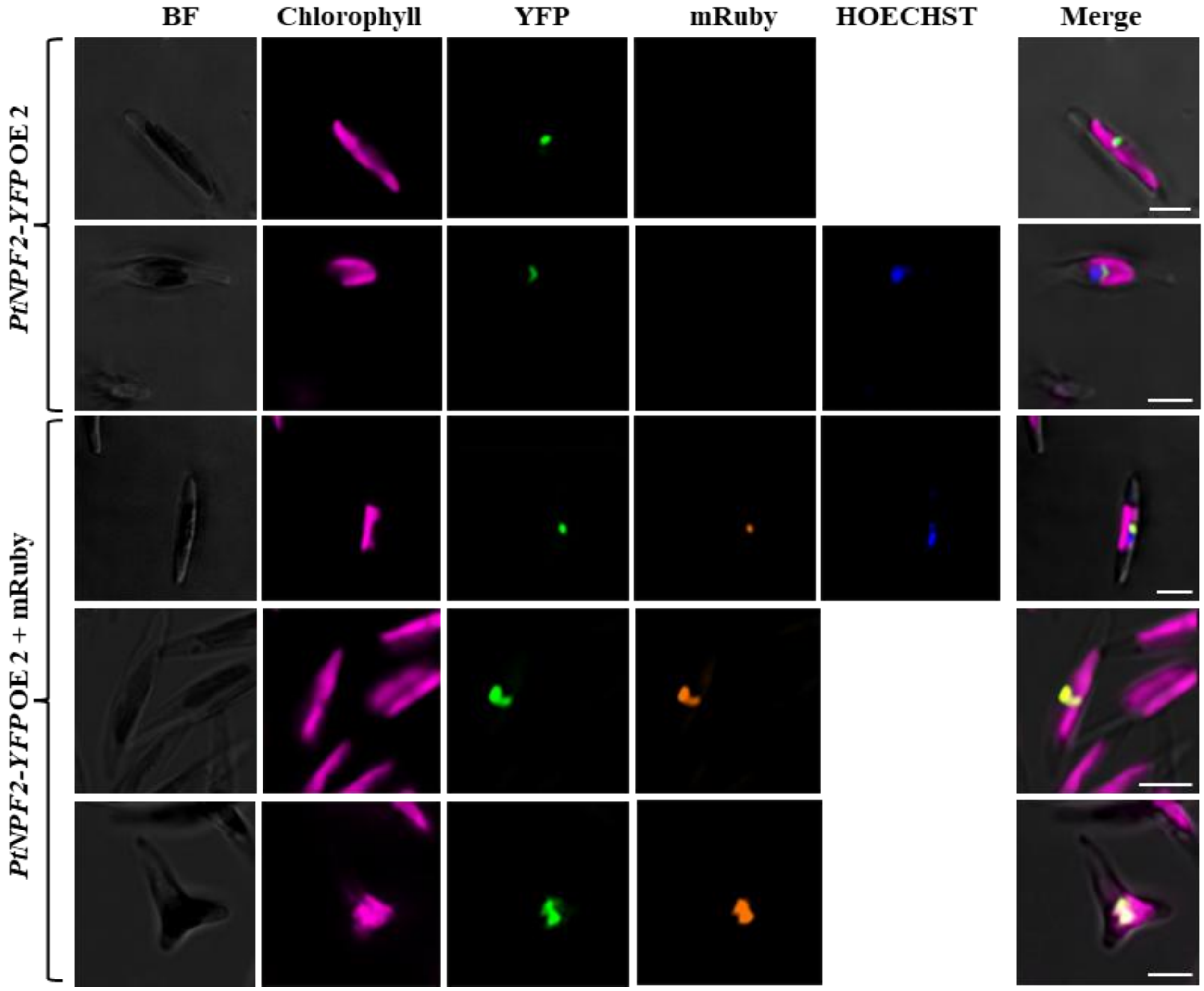

Fig S4. Growth curves of *P. tricornutum* wild-type, PtNPF2-YFP overexpressing, and knock-out strains. Growth curves of *P. tricornutum* wild-type (WT), two PtNPF2-YFP overexpressing (OE) strains, two *Ptnpf2* knock-out (KO) mutants and an additional *Ptnpf1* KO mutant, in different NO_3^- concentrations, N sources and pH conditions: A) pH 8 - 882 µM $NaNO_3$ (control pH and NO_3 concentration present in standard F/2 medium); B) pH 8 - 50 µM $NaNO_3$ as N starvation condition; C) pH 8 - 882 µM NH_4Cl and D) pH 8 - 882 µM urea as alternative N sources; E) pH 9 - 882 µM $NaNO_3$, F) pH 7 - 882 µM $NaNO_3$ and G) pH 6 - 882 µM $NaNO_3$ as different pH conditions; H) pH 7 - 882 µM NH_4Cl and I) pH 7 - 882 µM urea as combined low pH and alternative N sources; L) pH 8 - 882 µM KNO_3 as alternative combined salt in NO_3 compound. *In vivo* chlorophyll *a* fluorescence was used for measurements as proxy for growth. Error bars represent standard deviations (+/- SD) of three biological replicates. Statistical significance: * indicates $p < 0.05$; ** for $p < 0.01$ and * for $p < 0.001$.

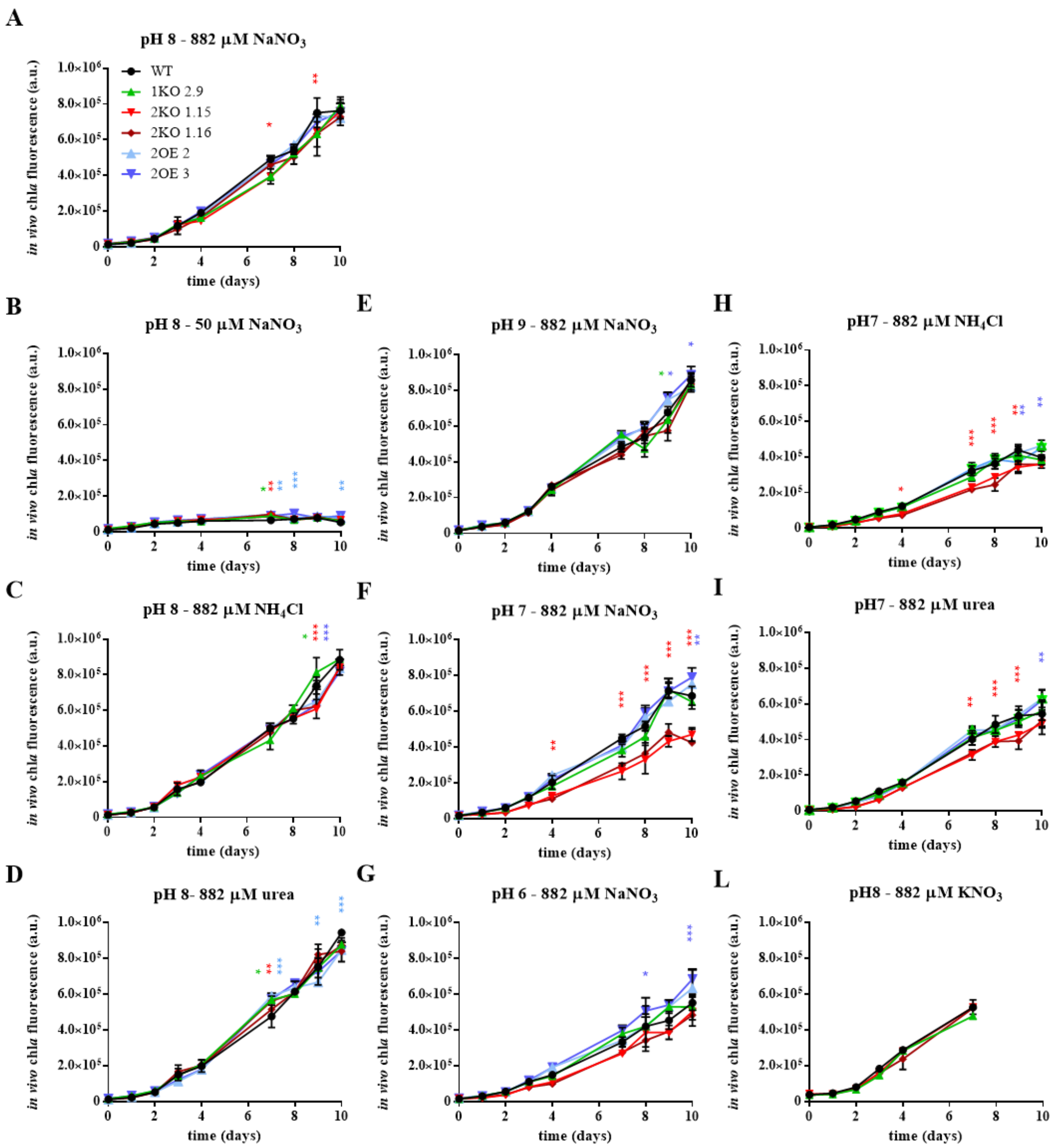

Fig S5. Pigment composition of *P. tricornutum* strains following pH shift. Pigment analysis performed on *P. tricornutum* strains, wild-type (WT) and *Ptnpf2* knock-out (KO) mutants 1.15 and 1.16, after 24 hours from pH shift. Chlorophyll *c*, β-carotene, Diadinoxanthin and Fucoxantin molar content, normalized on chlorophyll *a* molar content. Error bars represent standard deviations (+/- SD) of three biological replicates. Statistical significance: * indicates $p < 0.05$; ** for $p < 0.01$ and * for $p < 0.001$.

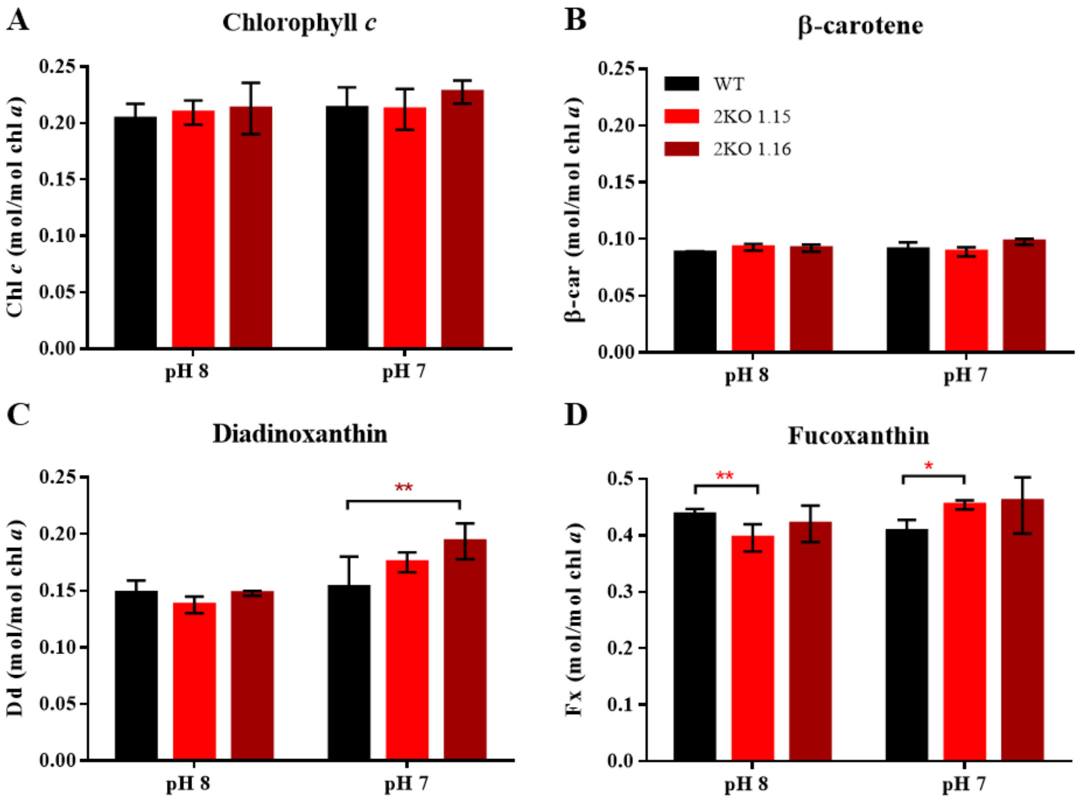

Fig S6. Overview of the transcriptome of *P. tricornutum* strains after the shift from normal to low pH. *P. tricornutum* wild-type (WT) and *Ptnpf2* knock-out (KO) mutant 1.15 after 24 hours from shift from pH 8 to pH 7, performed in 882 µM $NaNO_3$. A) Principal component analysis (PCA) shows the effect of strain and pH on the two axes. B) Numbers of differentially expressed genes (DEGs) for all comparisons. In the "KO vs WT" comparison “WT” was set as reference, while in "pH7 vs pH8" comparison “pH8” was set as reference. C) Venn diagrams and clustering analysis of transcriptomic results, combining single comparisons to find strain specific genes regulated in low pH. D) Total functional enrichment analysis of DEGs in *P. tricornutum*: Y-axis indicates enriched pathways, upper X-axis represents the enrichment score, represented by dots, while lower X-axis displays the number of DEGs, shown as bars.

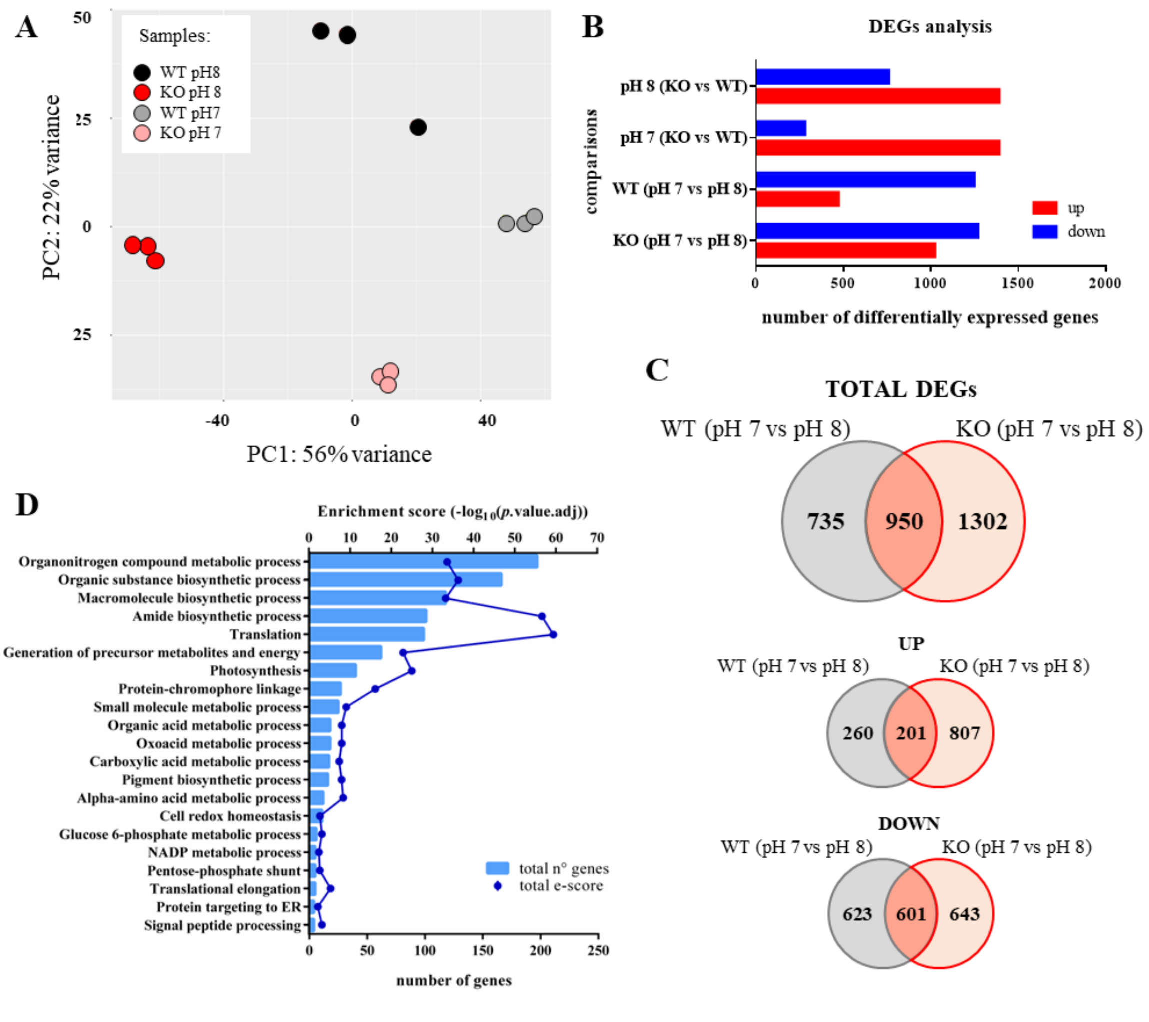

Fig. S7. Gene expression differences between *P. tricornutum* strains after the shift from normal to low pH. *P. tricornutum* wild-type (WT) and *Ptnpf2* knock-out (KO) mutant 1.15 after 24 hours from shift from pH 8 to pH 7, performed in 882 µM $NaNO_3$. Heatmap of the top 40 differentially expressed genes (DEGs) in the *P. tricornutum* transcriptome. The intensity of the orange/purple colours represents the normalized expression level. The rows have been sorted according to the gene clustering tree following Pearson correlation. The left-hand bar is colour-coded according to the functions of the genes.

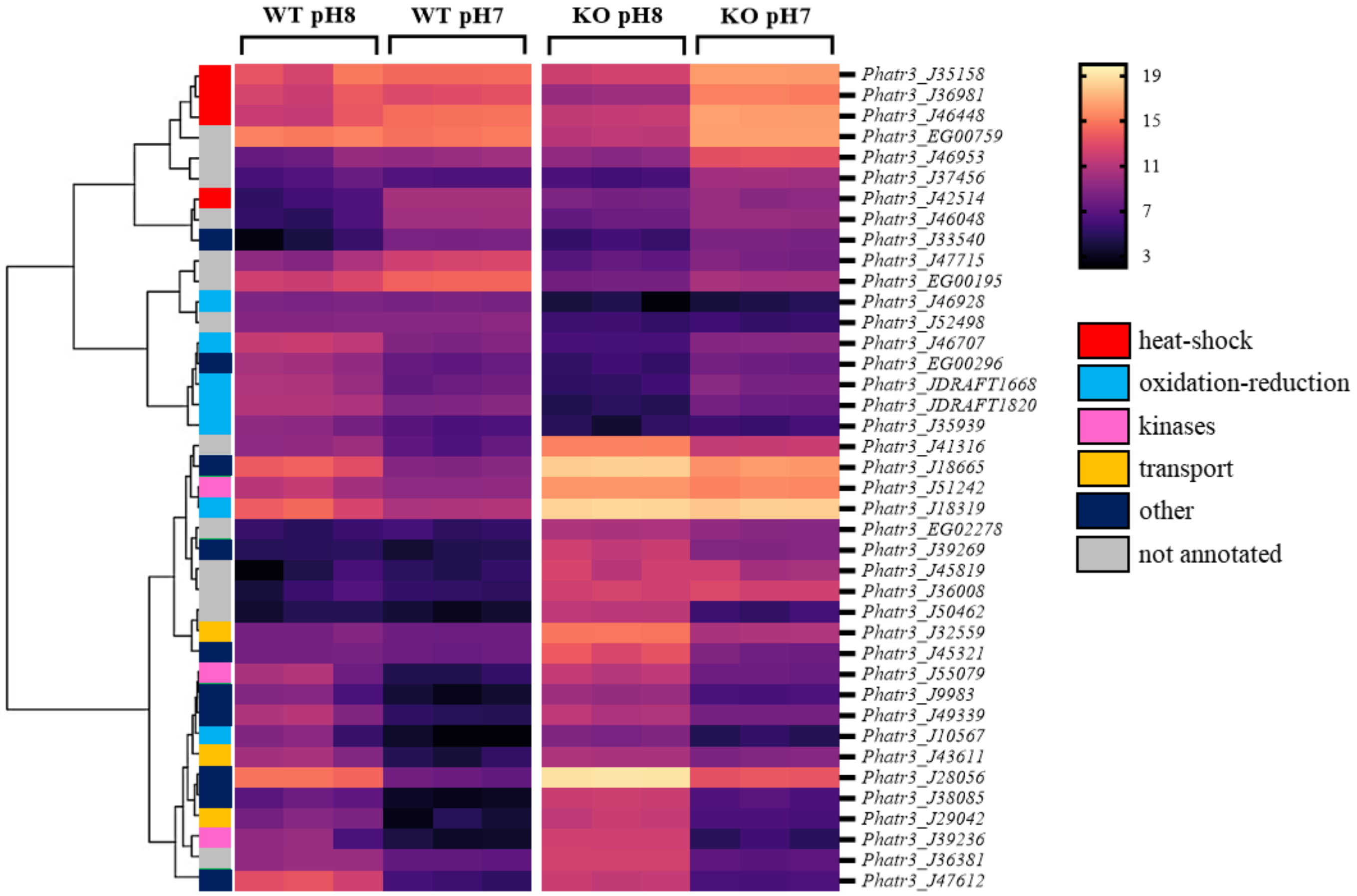

Fig. S8. Sequencing of the off-target gene Pt48498 on the *Ptnpf2* knock-out (KO) strain 1.16. As an off-target gene was found to be mutated in *Ptnpf2* KO strain 1.15, the same gene was sequenced in the *Ptnpf2* KO strain 1.16 genome, to exclude that this off-target could be responsible of the observed growth and photosynthetic phenotype shared by the two mutants. No mutations were found for gene Pt48498 in strain 1.16. A) PCRs on gDNA, B) PCRs on cDNA and C) sequencing of different wild-type and *Ptnpf2* knock-out strains.

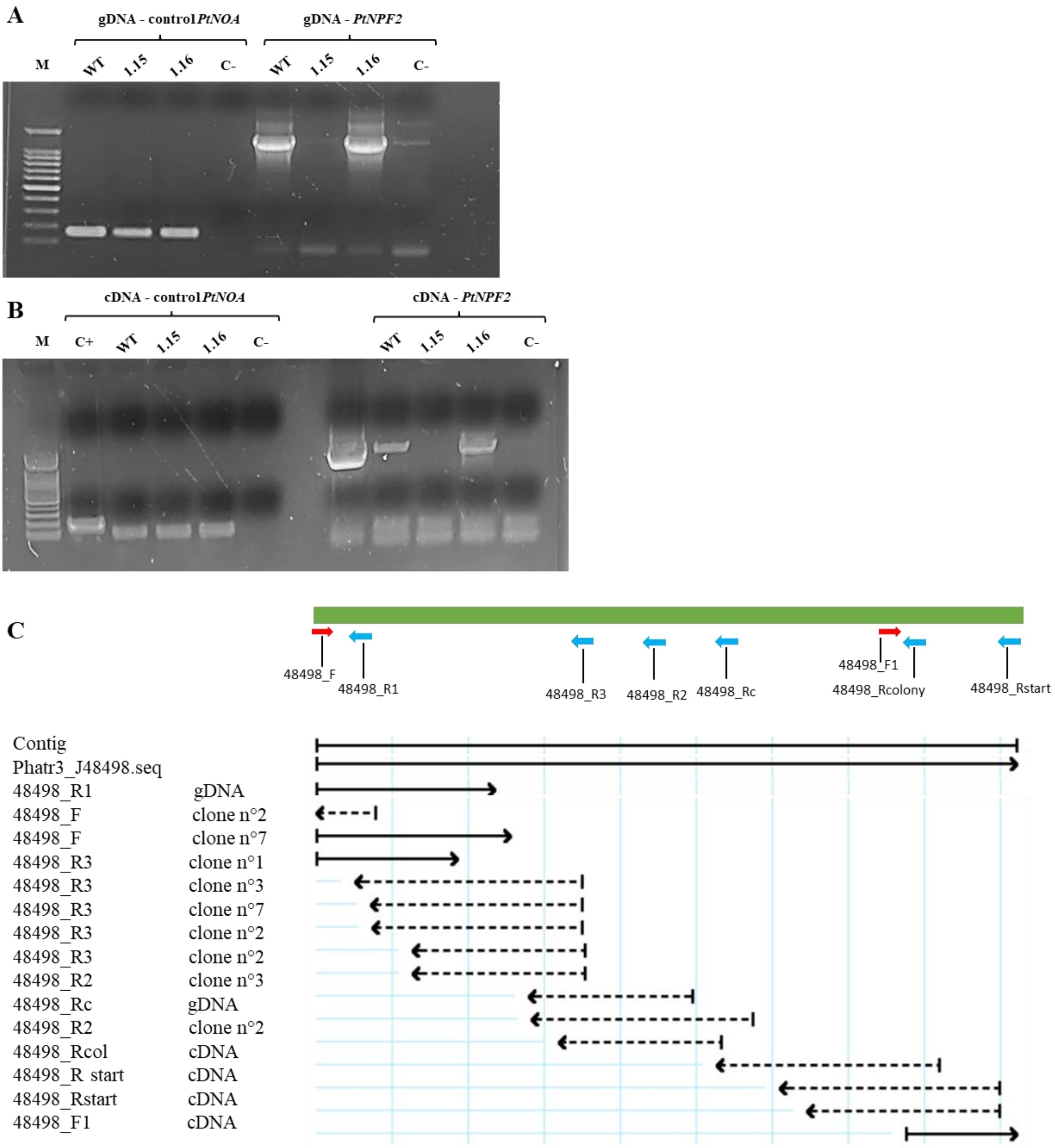

Fig. S9. Representation of physiological and transcriptional changes in *P. tricornutum* wild-type (WT) and *Ptnpf2* knock-out (KO) strains exposed to normal and low pH. A) Changes occurring during pH acidification in wild-type cells, meaning in cells shifted from pH 8 to pH 7. B) Changes occurring in the *Ptnpf2* KO mutant compared to the wild-type, in normal pH (pH 8). C) Changes occurring in the *Ptnpf2* KO mutant compared to the wild-type, after the shift to low pH. Different colours indicate different intracellular compartments, in particular cytosol and chloroplast. PtNPF2 is represented as transporter putatively located on the periplastidial compartment (PPC), between chloroplast membranes, when present in the wild-type cells. Normal font is used for physiological parameters, italics for the transcriptional differences. Arrows indicate the change direction, with red arrows for induction or upregulation and blue ones for inhibition/reduction or downregulation. PSII eff: PSII photosynthetic efficiency; NPQ: Non-Photochemical Quenching; *LHCs*: Light-harvesting related genes; *Xant*: xanthophyll cycle related genes; *ABCs*: genes encoding for ATP-binding cassettes; *HSPs*: genes encoding for Heat-shock proteins. This figure was created in BioRender (https://BioRender.com/m9jv1yc).

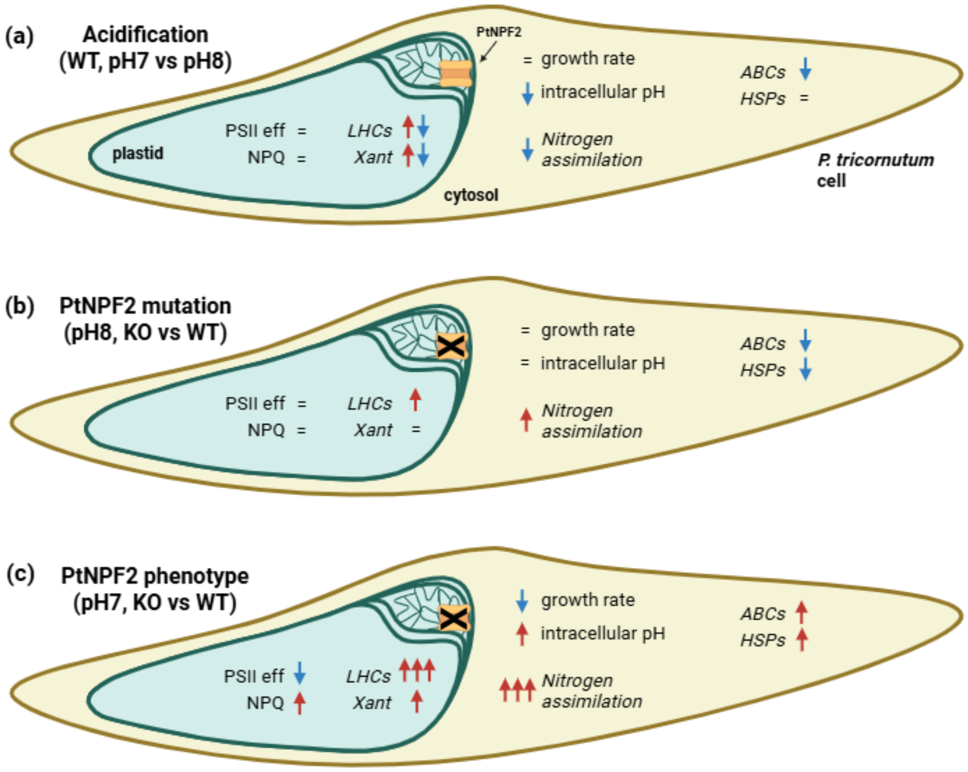

Fig. S10. Analysis of nitrate and dipeptide uptake by PtNPF2 in *Xenopus laevis* oocytes. A) Non injected, AtNPF6.3 (positive control) or PtNPF2-expressing oocytes were incubated in bathing medium containing 30 mM ^{15}N-labeled nitrate at different pH to search for ^{15}N-nitrate accumulation. B) Non injected, AtNPF8.1 (positive control) or PtNPF2-expressing oocytes were incubated in bathing medium containing 10 mM ^{13}C-labeled LEU-LEU at different pH to search for ^{13}C-LEU-LEU accumulation. Data are mean +/- SD (n = 5-10 oocytes). Statistical significance: ** indicates $p < 0.01$ and *** for $p < 0.001$. C) AtNPF8.1 (positive control) and PtNPF2 current-voltage relationships (I-V curves) in 10 mM ALA-ALA and LEU-LEU at different pH. Mean value +/-SD in n=3-5 oocytes.

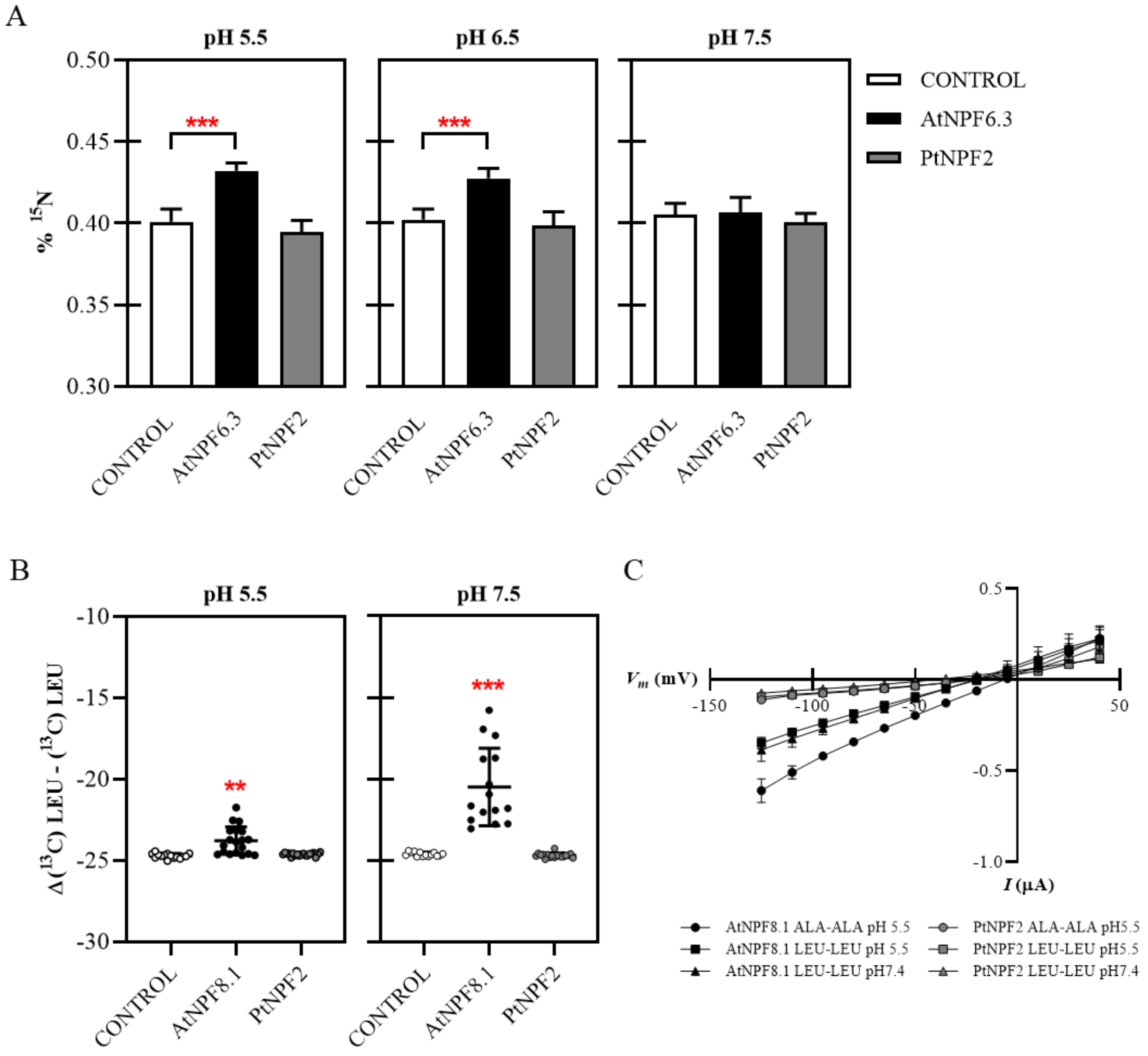

Supporting Tables.

Table S1. List of oligonucleotides information. crRNAs designed for CRISPR/Cas9 proteolistic transformation (with PAM sequence underlined), primers used for plasmid construction, primers used for overexpressing and knock-out mutants screening, and primers used for qPCRs.

Category	Name	Sequence
crRNAs	gAPT_1	5’-AAGCGTGGAATGCCTTTGAAGGG-3’ (-)
	gAPT_3	5’-CCAGGGCAATTGGTGGACCCAGG-3’ (-)
	gNPF2_a	5’-TGGTGTGCCTAGGAACAGGAGG-3’ (+)
	gNPF2_b	5’- TACGAGTGCAATCGATGTGACGG-3’ (-)
Primers for KO screening	PtAPT_fl_for	5’-ATGACGACAACCAACGGAAG-3’
	PtAPT_fl_rev	5’-TCAGTGTTCTTCGCCGTCATC-3’
	PtNPF2_ko_for	5’-ACGTTACCAAACAATCCTGTG-3’
	PtNPF2_ko_rev	5’-GTTGTGCTGAGTGAAGATCC-3’
	PtNPF2_exp_for	5’-TTACGTGATTGGCTTGTCCA-3’
	PtNPF2_fl_rev	5’-TCAAATCATTCTTCGCTC-3’
Primers for plasmids construction	Lhcf2pYPF_for	5’-GAATTCGATATCAAGCTTATCGAT-3’
	Lhcf2pYFP_rev	5’-GAATTCATGGTGAGCAAGGGCG-3’
	PmH4pGFP_for	5’-GCGGCCGCAACAACTACC-3’
	PmH4pGFP_rev	5’-AGGCCTCTTGTACAGCTCG-3’
	Lhcf2p_PtNPF2_for	5’-atcgataagcttgatatcgaattcATGAGTAGGAGAAGTTCTC-3’
	PtNPF2_YFP_rev	5’-cccttgctcaccatgaattcAATCATTCTTCGCTCTTC-3’
	GFP_PtNPF2_for	5’-acgagctgtacaagaggcctATGAGTAGGAGAAGTTCTC-3’
	PtNPF2_Lhcf1t_rev	5’-gaggtagttgttgcggccgcTCAAATCATTCTTCGCTC-3’
Primers for OE screening	Lhcf2p_for	5’-CGCCGTAAACAGCAAATCCT-3’
	PmH4p1_for	5’-AACAAAAGCTGGGTACCGGC-3’
	PtNPF2_exp_for	5’-TTACGTGATTGGCTTGTCCA-3’
	PtNPF2_exp_rev	5’-GGTCCGGCGTTATTAACAGA-3’
	GFP_for	5’-CCAGCAGAACACCCCCAT-3’
	GFP_rev	5’-AACTCCAGCAGGACCATGTG-3’
Primers for qPCR	PtNOA_exp_for	5’-CAGTTACTGACCCCCGAAGA-3’
	PtNOA_exp_rev	5’-AACGCACTTTCCGGTAGAGA-3’
	PtRPS_for	5’-GTGCAAGAGACCGGACATACC-3’
	PtRPS_rev	5’-CGAAGTCAACCAGGAAACCAA-3’
	PtNPF2_exp_for	5’-TTACGTGATTGGCTTGTCCA-3’
	PtNPF2_exp_rev	5’-GGTCCGGCGTTATTAACAGA-3’
	PtNRT2.1_rt_for	5’-TGGGATATTCAATTCTTGCGTCA-3’
	PtNRT2.1_rt_rev	5’-GCATGCCTCTTCAAGCTCAG-3’
	PtLHCX1_rt_for	5’-CCTTGCTCTTATCGGCTCTG-3’
	PtLHCX1_rt_rev	5’-ACGGTATCGCTTCAAAGTGG-3’
	PtVDR_rt_for	5’-GAAGCCGTTCGTCATCTTGC-3’
	PtVDR_rt_rev	5’-CGTCTTGCTCTTCGATGGGA-3’
	PtHSP2_rt_for	5’-TCGAGGCAAAATGGATCGCT-3’
	PtHSP2_rt_rev	5’-CCGCCTTCGACTCTACCATC-3’
	PtABC1_rt_for	5’-AGTGTGGTTGGCAGGGAAAA-3’
	PtABC1_rt_rev	5’-TGTTTGGGTTCCTCTACCGC-3’
	PtABC2_rt_for	5’-CTGGAACGACAAGGGTTCGA-3’
	PtABC2_rt_rev	5’-CTGACTGGTGGTCTTTCCCC-3’

Table S2. qPCRs performed on selected genes on *P. tricornutum* wild-type (WT) and *Ptnpf2* knock-out (KO) strains 1.15 and 1.16. Data represent Log_2 fold-changes (± SD, three biological replicates analysed) of the comparisons described: WT (pH7 vs pH8) indicates the conditions that determined pH-responsive genes, pH8 (KO vs WT) and pH8 (KO vs WT) indicate comparison between KO and wild-type grown at different pH for 24 hours. Considering the different sensitivity of RNA-seq and qPCR, data from the two different techniques are generally in accordance.

**Gene**	**WT (pH7 vs pH8) pH-response**		**pH8 (KO vs WT)**			**pH7 (KO vs WT)**		
	RNA-seq	***qPCR***	***KO 1.15 RNA-seq***	***KO 1.15 qPCR***	***KO 1.16 qPCR***	***KO 1.15 RNA-seq***	***KO 1.15 qPCR***	***KO 1.16 qPCR***
J26029 (NRT2)	-0.873 ± 0.006	-0.759 ± 0.077	+3.459 ± 0.001	+1.744 ± 0.463	+1.989 ± 0.614	+3.673 ± 0.001	+0.256 ± 0.061	+0.992 ± 0.194
J27278 (LHCX1)	-1.004 ± 0.001	+0.288 ± 0.079	+0.314 ± 0.004	+0.183 ± 0.047	+0.470 ± 0.144	-0.616 ± 0.001	-0.177 ± 0.026	+0.402 ± 0.072
J43240 (VDR)	-3.230 ± 0.001	-1.164 ± 0.074	+0.594 ± 0.001	+0.368 ± 0.111	-0.229 ± 0.061	+1.999 ± 0.001	+1.760 ± 0.404	-1.400 ± 0.045
J54656 (HSP2)	-2.050 ± 0.001	-2.178 ± 0.074	-1.735 ± 0.001	-1.279 ± 0.144	-5.466 ± 0.002	+4.059 ± 0.001	+2.353 ± 1.412	+0.075 ± 0.042
J32559 (ABC1)	-0.580 ± 0.004	+2.081 ± 0.630	+6.478 ± 0.001	+1.848 ± 0.488	+1.887 ± 0.589	+2.883 ± 0.001	+2.083 ± 0.470	-0.541 ± 0.074
J46086 (ABC2)	-2.791 ± 0.001	-1.863 ± 0.054	-2.728 ± 0.001	+0.119 ± 0.033	-2.805 ± 0.018	+2.842 ± 0.001	+1.837 ± 0.645	+0.128 ± 0.046

Table S3. Complete overview of the entire transcriptome of *P. tricornutum* wild-type (WT) and *Ptnpf2* knock-out (KO) strain 1.15. Transcriptomic analysis performed before (pH 8) and after 24 hours from the pH shift (pH 7). Information includes gene IDs, annotations, KEGG functions, Log_2 Fold-Changes (LFCs) and *p* values adjusted for all genes regulated in the comparisons WT (pH7 vs pH8), KO (pH7 vs pH8) as well as in the comparisons pH8 (KO vs WT) and pH7 (KO vs WT). FLCs above ±1 and *p* values adjusted < 0.05 were considered significant.

Supporting Methods.

Methods S1. Growth experiments.

Phaeodactylum tricornutum wild-type (WT), two PtNPF2-YFP overexpressing (OE) strains, two *Ptnpf2* knock-out (KO) mutants and an additional *Ptnpf1* KO mutant, growing axenically to mid-exponential phase were diluted to 2×10^5 cells ml^{-1} and transferred in buffered F/2 medium without silica (Guillard, 1975) in different conditions: at pH 8 with 882 µM $NaNO_3$ (control pH and NO_3 concentration present in standard F/2 medium); at pH 8 with 50 µM $NaNO_3$ as N starvation condition; at pH 8 with different N sources, namely 882 µM NH_4Cl or 882 µM urea. Other conditions included different pH: pH 9, pH 7 and pH 6 with 882 µM $NaNO_3$. Also, different pH and N sources combinations were tested: pH 7 with 882 µM NH_4Cl and pH 7 with 882 µM urea. To test a different salt, pH 8 medium with 882 µM KNO_3 was used as alternative combined salt in NO_3 compound. Each condition was set up in triplicate. The cell concentration was evaluated through *in vivo* chlorophyll *a* fluorescence, a proxy for growth, measured through a multifunctional monochromator-based microplate reader (Infinite™ M1000 Pro; Tecan, Palm Springs, CA, USA), and confirmed through the flow cytometer BD FACSVerse™ (BD Biosciences, San Jose, CA, USA).

Methods S2. Heterologous expression in *Xenopus laevis* oocyte.

For expression in *Xenopus laevis* oocytes, *PtNPF2* was cloned into vector pGEMKN (kindly provided by Dr. Dirk Becker, University of Wurzburg), which is a derivate of the previously described pGEMHE (Liman *et al.*, 1992). Plasmids were linearized and transcribed *in vitro* with HiScribe T7 ARCA mRNA Kit (New England Biolabs), following the manufacturer's protocol. Oocytes were obtained and injected as previously described (Lacombe & Thibaud, 1998). Two days after injection with cRNA, injected and negative control (water-injected) oocytes were incubated 2 hours in 2 mL of ND96 medium (2 mM KCl, 96 mM NaCl, 1 mM $MgCl_2$, 1.8 mM $CaCl_2$, 5 mM MES/Tris, pH 5.5) containing 30 mM of $K^{15}NO_3$ (atom % ^{15}N abundance: 99.9%, Sigma-Aldrich) or 10 mM ^{13}C-LEU-LEU to test influx activity (Léran *et al.*, 2015). For pH experiments, ND96 solution was adjusted at pH 5.5 (MES), 6.5 (MES) or 7.5 (HEPES). Oocytes were then washed 5 times in 15 mL of ND96 medium (pH 5.5) at 4°C and dried at 65°C for 24 hours. Oocytes were then analysed for ^{15}N or ^{13}C abundance by determining $^{14}N/^{15}N$ ratio and $\Delta^{13}C$ in individually dried oocytes with Continuous Flow Mass Spectrometry, using a VarioPyroCube elemental analyser (Elementar, UK) coupled with an IsoPrime Precision mass spectrometer (Elementar, UK).

Electrophysiological measurements were made 3 days after cRNA injection in oocytes, using a two-electrode voltage-clamp technique. RINGER (2 mM KCl, 115 mM NaCl, 1 mM MgCl2, 1.8 mM CaCl2, 5 mM HEPES (pH 7.4) or 5 mM MES (pH5.5)) bathing solution with or without 10 mM dipeptides (LEU-LEU or ALA-ALA) were used. Current-passing and voltage-recording electrodes were filled with 3 M KCl and had 0.5-1.5 MW tip resistance in 100 mM KCI. The voltage-clamp amplifier was a Geneclamp 500B (Axon, USA). Voltage-pulse protocols, data acquisition and data analysis were performed using the pClamp program suite (Axon Instruments, USA). Both membrane potential and current were recorded. Correction was made for voltage drop through the series resistance of the bath and the reference electrode by using a voltage-recording microelectrode in the bath close to the oocyte (the potential of the bath electrode was subtracted from the one of intracellular electrodes in the amplifier, allowing a real-time series resistance correction). All experiments were performed at room temperature (20-22°C).

Supporting References.

Lacombe B, Thibaud J-B. **1998**. Evidence for a Multi-ion Pore Behavior in the Plant Potassium Channel KAT1. *The Journal of Membrane Biology* **166**: 91–100.

Léran S, Edel KH, Pervent M, Hashimoto K, Corratgé-Faillie C, Offenborn JN, Tillard P, Gojon A, Kudla J, Lacombe B. **2015**. Nitrate sensing and uptake in Arabidopsis are enhanced by ABI2, a phosphatase inactivated by the stress hormone abscisic acid. *Science Signaling* **8**: ra43–ra43.

Liman ER, Tytgat J, Hess P. **1992**. Subunit stoichiometry of a mammalian K+ channel determined by construction of multimeric cDNAs. *Neuron* **9**: 861–871.
